# Temperature, moisture and freeze–thaw controls on CO_2_ production in soil incubations from northern peatlands

**DOI:** 10.1038/s41598-021-02606-3

**Published:** 2021-12-01

**Authors:** Eunji Byun, Fereidoun Rezanezhad, Linden Fairbairn, Stephanie Slowinski, Nathan Basiliko, Jonathan S. Price, William L. Quinton, Pascale Roy-Léveillée, Kara Webster, Philippe Van Cappellen

**Affiliations:** 1 Ecohydrology Research Group, Department of Earth and Environmental Sciences and Water Institute, University of Waterloo, Waterloo, ON Canada; 2 Environment and Climate Change Canada, Toronto, ON Canada; 3 Department of Biology and Vale Living With Lakes Centre, Laurentian University, Sudbury, ON Canada; 4 Department of Geography and Environmental Management, University of Waterloo, Waterloo, ON Canada; 5 Cold Regions Research Centre, Wilfrid Laurier University, Waterloo, ON Canada; 6 Université Laval, Quebec City, QC Canada; 7 Canadian Forest Service Great Lakes Forestry Centre - Natural Resources Canada, Sault Ste Marie, ON Canada

**Keywords:** Carbon cycle, Biogeochemistry, Environmental sciences, Boreal ecology, Climate-change ecology, Wetlands ecology

## Abstract

Peat accumulation in high latitude wetlands represents a natural long-term carbon sink, resulting from the cumulative excess of growing season net ecosystem production over non-growing season (NGS) net mineralization in soils. With high latitudes experiencing warming at a faster pace than the global average, especially during the NGS, a major concern is that enhanced mineralization of soil organic carbon will steadily increase CO_2_ emissions from northern peatlands. In this study, we conducted laboratory incubations with soils from boreal and temperate peatlands across Canada. Peat soils were pretreated for different soil moisture levels, and CO_2_ production rates were measured at 12 sequential temperatures, covering a range from − 10 to + 35 °C including one freeze–thaw event. On average, the CO_2_ production rates in the boreal peat samples increased more sharply with temperature than in the temperate peat samples. For same temperature, optimum soil moisture levels for CO_2_ production were higher in the peat samples from more flooded sites. However, standard reaction kinetics (e.g., *Q*_10_ temperature coefficient and Arrhenius equation) failed to account for the apparent lack of temperature dependence of CO_2_ production rates measured below 0 °C, and a sudden increase after a freezing event. Thus, we caution against using the simple kinetic expressions to represent the CO_2_ emissions from northern peatlands, especially regarding the long NGS period with multiple soil freeze and thaw events.

## Introduction

Surface air temperatures in northern high latitude regions are increasing two to six times faster than the global average^1^, a trend also observed in Canada^2^. Climate models project that this rate of warming will continue through the twenty-first century, with the greatest warming occurring during the fall, winter and spring period, which is generally non-growing season (hereafter ‘NGS’)^3^. Given that microbially mediated soil organic carbon (SOC) mineralization (or heterotrophic soil respiration) responds positively to increasing temperatures, warming during the NGS could substantially alter the carbon balance of high latitude soils. For example, arctic tundra ecosystems might already be shifting from annual carbon sinks to sources due to increasing NGS carbon emissions^4–7^. The extent to which such shifts apply to a wider spectrum of cold region soil carbon pools remains uncertain, however^8,9^.

In addition to the temperature-dependent slowing down of microbial metabolic activity, soil organic matter decomposition during the NGS can also be inhibited by soil freezing, which limits the supply of molecular oxygen (O_2_) and the availability of liquid water, even if there are sufficient organic substrates remaining from the growing season. Especially where the soil is poorly drained, a portion of the growing-season plant CO_2_ fixation is therefore not mineralized^10^. This leads to the accumulation of organic peat layers that can be preserved for up to several millennia^11^. The resulting northern peatlands have played a substantial role in storing atmospheric CO_2_ since the last deglaciation^12,13^. Nevertheless, recent rapid climate warming, as well as direct human encroachment of peatlands, threaten to release this globally important carbon pool into the atmosphere. While Canada has the second largest areal coverage of peatland^14^, much of its southern peatlands have already been lost to agriculture and impacted by other anthropogenic disturbances^15^. In Canada’s north (i.e., above 50°N), ongoing ecological and biogeochemical changes driven by climate warming are affecting the carbon storage potential of remaining natural peatlands.

Recent advances in data synthesis and modeling have highlighted the sensitivity of global soil respiration to climate change^16^, with significant implications for NSG soil carbon emissions in cold regions^8^. Nonetheless, the parameterization and calibration of process models used to simulate future trajectories of soil carbon-climate feedbacks remain poorly constrained when it comes to winter season carbon losses^8^. For example, a new version of the Canadian Model for Peatlands (CaMP v2.0) has been developed for inclusion into the national carbon budget estimations^17^. However, the model integrates soil carbon emissions over annual time steps and, hence, does not account for seasonal variations in peat decomposition kinetics, which in turn produces unaccountable uncertainties in model predictions under changing climate conditions^14,18–20^.

One way to improve the representation of NGS soil carbon dynamics, is to incorporate variable reaction parameters that reflect the seasonal changes of key soil environmental variables, in particular soil temperature and moisture^21^. For example, the temperature sensitivity coefficient (*Q*_10_) has been widely used as a simple numerical parameter to correct rates of soil carbon mineralization and other microbially-controlled transformation processes^22–25^. The *Q*_10_ approach assumes an exponential increase in the reaction rate with increasing temperature, and a typical *Q*_10_ value of 2 implies that the rate doubles with every 10 °C increase in the ambient temperature^24^. Most land surface and dynamic vegetation models use the constant *Q*_10_ values, but recent findings suggest that the *Q*_10_ required to temperature-correct CO_2_ emissions may be higher (i.e., *Q*_10_ > 2) in cold climate regions^26–29^, likely because of the interplay of multiple factors affecting soil physiochemical properties (e.g., those accompanying pore water freeze and thaw) during the NGS^20,30^.

To date, a considerable amount of soil carbon *Q*_10_ values have been reported from field and laboratory studies, thus enabling global syntheses^22,31^. However, a relatively limited number of studies have focused on the warming response of organic-rich wetland and peatland soils despite their disproportionately large share of the global SOC stock^10,32^, and considering the rapid NGS changes in high latitudes^4–8^. In addition to the effect on the temperature regime of northern peatland soils, rapid climate warming is also driving changes in landscape hydrology and, thus, in the moisture status of the soils^21,33^. Thus, latitudinal, and seasonal variations in soil temperature and moisture should be considered when estimating SOC mineralization rates. Without consideration of such variations, projections of future annual peatland CO_2_ emissions will be fraught with uncertainty, especially at higher latitudes where the impacts of climate change are expected to be most pronounced in the winter and shoulder seasons (fall and spring) compared to summer^2^.

Because it is challenging to have regular or continuous access to multiple remote peatland locations in wintertime to perform in situ flux measurements, laboratory experiments with field samples can help with the calibration of model parameters (for example, *Q*_10_ values as discussed above). The lab environment allows one to perform measurements of CO_2_ production rates under simulated NGS conditions by controlling key environmental factors, such as temperature, moisture content and freezing-induced changes in the accessibility of organic substrates^20,34–37^.

In this study, we conducted laboratory incubations with peat samples of different depth intervals retrieved at seven Canadian peatland locations in two climate zones (boreal and temperate). We measured CO_2_ release fluxes under variable moisture levels across a temperature sequence ranging between − 10 and + 35 °C. The resulting CO_2_ production rates were fitted to equations describing the observed moisture and temperature trends. Our objectives were to (1) delineate systematic differences in the *Q*_10_ of CO_2_ production between sites, and relate them to the ecoclimate, sample depth and moisture content, (2) identify the optimum moisture levels for CO_2_ production, and (3) assess the impact of a soil freezing event on the CO_2_ production during the event and following thaw. We also explored the use of the Macromolecular Rate Theory (MMRT; see Alster et al.^38^ and references therein) as a general framework to describe the measured CO_2_ production rates.

## Materials and methods

### Field peat sampling

**Figure 1:**
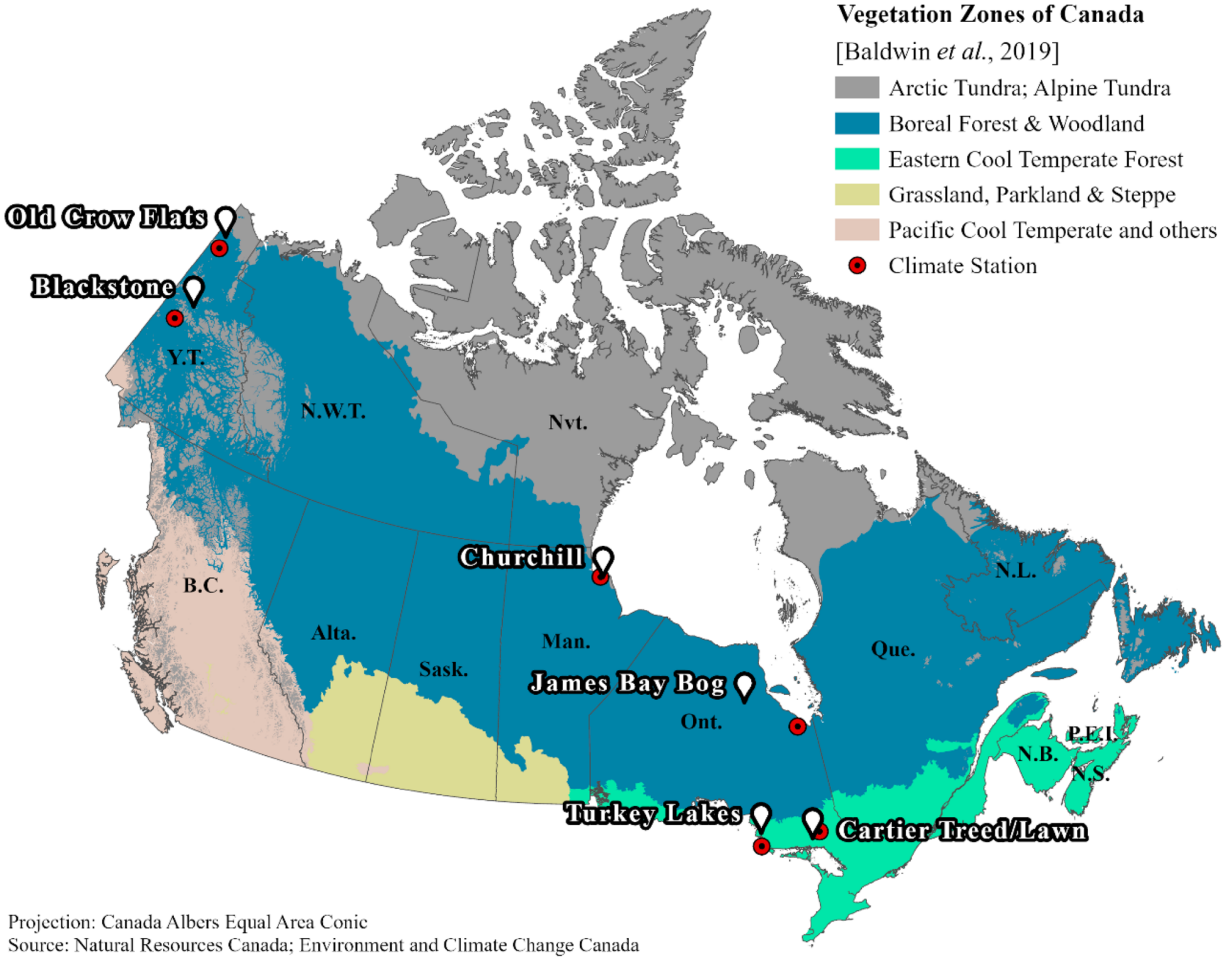
The locations of peatland sites across different vegetation (ecoclimate) zones of Canada (Baldwin et al*.* 2019) with the nearest weather stations (circles) for which Canadian Climate Normals 1981–2010 data are available (Environment and Climate Change Canada). The map was created using ArcGIS Pro (Esri). The site names correspond to the information given in Table 1.

Peat samples were collected from three peatland sites in “Eastern Cool Temperate Forest” (‘temperate’ hereafter) and four sites in “Boreal Forest & Woodland” (‘boreal’ hereafter) biogeoclimatic vegetation zones (‘ecoclimate’ hereafter), based on the classification of Baldwin et al.^39^ (Vegetation Zones of Canada: a Biogeoclimatic Perspective—Level 1; see Table 1 and Fig. 1). We chose this recent land classification map over the other commonly used national terrestrial ecozones classification^14^ because it closely parallels the Köppen-Geiger climate classes^40^ for the sampling sites (see Supplementary Figure S1). At each field location, peat samples were collected from depth intervals 0–10, 10–20 and 20–30 cm with a wide-toothed saw. The top 5 cm was removed to minimize large litter debris. All the samples were collected wet, immediately stored in a cooler and transported to the University of Waterloo. Upon arrival, the samples were placed in an anaerobic chamber (Mandel Scientific Anaerobic Chamber, AC11-074) and remained uncovered at room temperature (25 °C) under anoxic atmosphere for two weeks while being hand-mixed at regular daily intervals. Next, the total porosity, bulk density and moisture content of the peat samples were determined gravimetrically from the saturated mass, oven-dried mass (2–10 g wet soil at 80 °C for 24 h) and original volume of the sample, following the methods of Gardner^41^.

**Table 1:** Peatland sampling site information. Peat samples collected from each of the 10-cm depth intervals were analyzed for dry bulk density (BD, g cm^−3^) and organic carbon content (OC, %).

Site	Latitude (°N)	Longitude (°W)	Peatland type	Ecoclimate*	MAT^†^ (°C)	Description^‡^	Depth (cm)	BD (g cm^−3^)	OC^§^ (%)
Old Crow Flats, Yukon	68.1148	140.05085	Sphagnum tundra	Boreal	− 8.27	Open vegetation cover with *Sphagnum* moss, *Eriohorum* tussocks, *Ledum decumbens* and *Rubus chamaemorus*. Samples were collected from moss cover	0–10 10–20	0.055 0.098	46.4 46.0
Blackstone Uplands, Yukon	64.91937	138.28308	Tussock tundra	Boreal	− 4.1	Poorly drained low-grade terrain with red *Sphagnum* moss, *Eriphorum* tussocks, and some lichen (*e.g., Flavocetraria nivalis*). Ground is slightly hummocky, and sampling was from the hummock top	0–10 10–20	0.067 0.088	46.1 45.5
Churchill, Manitoba	58.72247	93.8477	Lichen tundra	Boreal	− 6.47	Polygonal peat plateau. Plateau surface forms slightly raised ground dissected by ice wedge troughs. Lichens cover with some sedges, ericaceous shrubs (*Vaccinium vitis-idea, Ledum decumbens*) and *Rubus chamaemorus*	0–10 10–20 20–30	0.111 0.151 0.135	48.6 49.1 48.8
James Bay Bog, Ontario	52.73733	83.97382	Sphagnum bog	Boreal	− 0.45	Raised bog, hummocky with some lawn. Ground cover dominated by moss, mostly *Sphagnum fuscum*, but in places up to 50% *Cladonia rangiferina*, with 5 to 50% vascular plants mostly *Rubus Chamaemorus* and *Chamaedaphne calyculata*, and sparse tree cover, mostly *Picea mariana*	0–10 10–20 20–30	0.087 0.062 0.070	46.1 45.2 47.8
Turkey Lakes, Ontario	47.04809	84.40709	Hardwood swamp	Temperate	4.73	Mixed stands of *Fraxinus nigra*, *Thuja occidentalis*, *Acer rubrum*, yellow birch and *Larix laricina*. Understories with the seedlings and saplings of the trees, various ferns, herbs (*e.g., Caltha palustris*, *Carex trisperma*, and *Impatiens capensis*) and a mix of feather and Sphagnum mosses	0–10 10–20 20–30	0.143 0.329 0.067	44.5 44.5 41.4
Cartier Treed, Ontario	46.3976	81.3123	Treed poor fen	Temperate	4.1	Pronounced hummock-hollow microtopography with continuous Sphagnum moss understory, moderately dense shrub layer (*Rhododendron groenlandicum, Chamaedaphne calyculata*), sparse sedges in hollows, and 5–10 m tall, well-spaced/open canopy *Picea mariana* trees. Samples were collected from hummocks. Moss water pH was ~ 4.4 and water table 80 cm below the surface at time of sampling	0–10 10–20 20–30	0.132 0.118 0.061	46.2 47.4 48.1
Cartier Lawn, Ontario	46.3777	81.312	Open poor fen	Temperate	4.1	A thick floating mat (> 5 m) in a terrestrializing pond adjacent to the treed fen site with little microtopography, continuous Sphagnum moss cover, and moderately dense (~ 35% cover) *Eriophorum spissum*, sparse low shrubs (*Chamaedaphne calyculata, Vaccinium oxycoccos*), and small (0.5–1 m) *Larix laricina*. Pore water pH was ~ 4.3 and water table 5 cm below the surface at time of sampling	0–10 10–20 20–30	0.161 0.052 0.100	46.2 46.7 48.8

*According to Vegetation Zones of Canada: a Biogeoclimatic Perspective—Level 1^39^.

^†^Mean annual temperature data from 1981–2010 Climate Normals, Government of Canada (see weather station names in Supplementary Table S2).

^‡^Turkey Lakes site description from Webster et al.^42^.

^§^Samples are combusted at 600 °C in an elemental analyzer.

### Laboratory incubations and CO_2_ production rates

After the two weeks of chamber-drying, the peat samples were homogenized and divided into five subsamples of approximately equal mass and volume. These subsamples were placed in five 500-mL glass mason jars, filling less than half the space. The initial dry masses of peat varied by site and depth, from ~ 120 to ~ 600 g (or ~ 24 to ~ 120 g per jar), with lower masses for the drier and lower density peat samples (e.g., those from the shallowest depth intervals). One in each of the five-jar series was incubated at the gravimetric moisture content of the peat remaining after the two weeks of chamber-drying. The moisture contents of the peat in the other four jars were adjusted by adding an artificial water solution prepared based on the chemical composition of the soil water collected at the corresponding site (Supplementary Table S1). The moisture contents were calculated as water-filled pore space (%WFPS), based on the measured gravimetric moisture, bulk density and porosity, plus the volume of water solution added to the jar. We aimed at %WFPS ranging from the gravimetric moisture content of the peat up to 100%. Thus, each jar and the contained peat corresponded to a given site, depth interval, and moisture condition (e.g., Blackstone, 0–10 cm, 100% WFPS).

The jars were incubated with their lids closed and, therefore, water loss and changes in soil moisture were assumed to be negligible. The jars were incubated in an environmental chamber (Percival I-41NL XC9) which was cycled through a series of 12 sequential temperature settings of 25, 35, 25, 15, 10, 1, − 2, − 5, − 10, − 2, 5 and 25 °C. Note that the incubation temperature range was not restricted for a given sample location/depth to the site-specific in-situ soil temperatures. Rather, we imposed the same temperature range and trajectory (including one freezing event) for all the samples to assess the temperature effect, separate from other site- and soil-specific variables. Specifically, the incubation started at room temperature 25 °C, that is, the same temperature under which the peat samples were dried in the anoxic chamber. Thus, the peat samples adjusted to the aerobic and variable moisture conditions without experiencing a sudden temperature change when transferred out of the anoxic chamber. At the high temperature end, 35 °C is usually near the optimum temperature for soil microbial activity. By extending the temperature to the optimum value, we expected a more robust curve-fitting analysis than for a smaller temperature range^38^. Then, we purposely included repeated measurements at 25 °C to check for potential long-term drifts in the rates of CO_2_ production due, for example, to carbon substrate depletion or an irreversible shift in the microbial community structure, as commonly observed in long-term incubations. The temperature in the incubation chamber was lowered up to − 10 °C, considering that soil temperature at 0.1 m depth in northern permafrost peatland have been shown to drop to as low as − 10 °C during the winter^43^.

At each setting, the temperature fluctuations were within ± 0.5 °C. The CO_2_ production rates were determined after 48 h of incubation at a given temperature using an automated multiplexer CO_2_-flux system (LI-8100 and LI-8150, LI-COR Biosciences, Lincoln, NE, USA). The rates of headspace CO_2_ concentration increase with time (*d*CO_2_/*dt*, μmol m^−2^ s^−1^) were measured following the method of Davidson et al.^44^, considering the variable headspace volume of the jars (Note: for more details of the measurement setup and procedure, see Rezanezhad et al.^45^). To compare CO_2_ production among the different peat subsamples, the rates were normalized to the dry weight of the peat in each jar and expressed in units of µmol g^−1^ h^−1^. Overall, our incubation experiment was not aimed at quantifying the in-situ CO_2_ emissions from northern peatlands, but to delineate the response of CO_2_ production rates to variable temperature and moisture, as well as to a freeze–thaw event.

### Fitting of CO_2_ production rates

For each jar experiment (corresponding to a given site, depth interval, and moisture content), the CO_2_ production rates measured across a range of temperatures (*R*_*T*_) were fitted to the following exponential equation ^25,46^ with temperature (*T*, °C) as the independent variable:

1 $${{R}_{T}=\alpha e}^{(\frac{ln{Q}_{10}}{10})T}$$

where $\alpha$ and ${Q}_{10}$ are fitting coefficients determined by the method of least squares (‘fitnlm’ function in MATLAB R2020b), and *R*_*T*_ are the CO_2_ production rates normalized to the dry weight of the peat. Equation (1) is closely related to the Arrhenius equation, which states that the rate constant, *k,* of a reaction can be described by:

2 $$k= A{e}^{-\frac{{E}_{a}}{RT}}$$

where *R* is the universal gas constant, ${E}_{a}$ is the (empirical) activation energy and *A* is called the frequency or pre-exponential factor. Assuming that, for a given jar experiment, the pool of organic matter being mineralized at the different temperatures remains the same, $\alpha$ should be linearly related to *A*. From Eqs. (1) and (2) it then follows that:

3 $${Q}_{10}= {e}^{\frac{10{E}_{a}}{RT(T+10)}}$$

which implies that using *Q*_10_ as a measure of the temperature sensitivity is equivalent to assuming the CO_2_ production rate follows the Arrhenius equation. Also, note that ${E}_{a}$ may vary with temperature, although for relatively small temperature ranges, as considered in our experiments, ${E}_{a}$ is usually treated as a constant^24^. Practically, the *Q*_10_ value represents the proportional rate increase in CO_2_ production for a 10 °C increase of temperature (i.e., ${Q}_{10}= {R}_{T+10}/{R}_{T}$) and thus provides a simple metric to express the temperature sensitivity of the soil CO_2_ production kinetics.

The Arrhenius equation predicts that a reaction rate (here as the CO_2_ production rate) always increases with increasing temperature. Microbially-mediated reaction processes, however, often exhibit a temperature optimum at which the rate reaches its maximum value. This feature is reproduced by the Macromolecular Rate Theory (MMRT) (e.g., ^47–50^) according to which the temperature dependence of enzyme-catalyzed reactions is given by:

4 $$k= {\frac{{k}_{B}T}{h}e}^{-\frac{{[\Delta }^{\ddagger }{H}_{{T}_{0}}+{\Delta }^{\ddagger }{C}_{p}(T-{T}_{0})]}{RT}+\frac{[{\Delta }^{\ddagger }{S}_{{T}_{0}}+{\Delta }^{\ddagger }{C}_{p}\left(lnT-ln{T}_{0}\right)]}{R}}$$

where the temperature $T$ is now expressed in degrees K (0 °C = 273.15 K), ${T}_{0}$ (K) is a reference temperature point set at a few degrees below the estimated optimum temperature, *R* is the universal gas constant, ${k}_{B}$ is Boltzmann's constant, and $h$ is Planck's constant.

Equation (4) has three fitting parameters: the changes in the standard enthalpy (${\Delta }^{\ddagger }{H}_{{T}_{0}}$), entropy (${\Delta }^{\ddagger }{S}_{{T}_{0}}$), and heat capacity (${\Delta }^{\ddagger }{C}_{p}$) for the activation reaction (i.e., the formation of the enzyme–substrate transition state), with the standard Gibbs energy of activation then given by: ${\Delta }^{\ddagger }G= {\Delta }^{\ddagger }H- {T\Delta }^{\ddagger }S$. Note that for a heat capacity change of activation equal to zero*, *${\Delta }^{\ddagger }{C}_{p}=0$*,* MMRT predicts an exponential temperature dependence of the CO_2_ production rate that is equivalent to that of the Arrhenius equation. Physically plausible deviations from this exponential dependence should yield values ${\Delta }^{\ddagger }{C}_{p}<0$. For further details on the application of MMRT to microbial reaction processes, see Alster et al.^38^ and references therein. In this study, we attempted to find a general relationship between the CO_2_ production rate (*k*) and temperature (*T*) using Eq. (2) and (4) with the fitting parameters determined by the method of least squares (‘fitnlm’ function in MATLAB R2020b).

Using the measurements at 25 °C across the range of experimental moistures, the CO_2_ production rates (${R}_{25^{~\circ} {\mathrm{C}}}$) were fitted to a second-order polynomial equation (e.g., ^20,36,51^)

5 $${R}_{25^{~\circ} {\mathrm{C}}}=a{x}^{2}+bx+c$$

where $x$ is the moisture content (% WFPS). Equation (5) was fitted to the measured rates using the ‘polyfit’ function in MATLAB R2020b. Note that ${R}_{25^{~\circ} {\mathrm{C}}}$ reaches its maximum value at $x= -b/2a$ (% WFPS), which therefore provides a measure of the optimum moisture condition for CO_2_ production.

### Statistical analyses

The statistical analyses were performed in R version 4.0.3 using the ‘rpart,’ ‘ggplot2,’ and ‘stats’ packages. A decision tree learning approach (function ‘rpart’) was used to identify the relative importance of ecoclimate, peat depth, and soil moisture content in predicting the *Q*_10_ values. The *Q*_10_ values were then summarized for each site and ecoclimate in box-and-whisker plots (function ‘ggplot’). One-way ANOVA (function ‘aov’) was performed to examine whether the *Q*_10_ values significantly varied from site to site, as well as between sites in the boreal versus temperate ecoclimate zone. Statistical differences between any two groups of samples were evaluated by performing t-tests (function ‘t-test’).

A simple linear regression analysis (function ‘lm’) was conducted to explore the apparent influence of local site climate conditions on the *Q*_10_ variance using the mean site *Q*_10_ values and the mean air temperatures recorded during the 1981–2010 Climate Normals at the nearest weather station (Supplementary Table S2). The annual mean site air temperature was chosen as a simple local climate proxy. Additionally, the mean annual air temperature range, calculated as the difference between the July (warmest) and January (coldest) mean temperatures, was also considered as a possible explanatory variable.

## Results

### Temperature dependence of CO_2_ production rates

**Figure 2:**
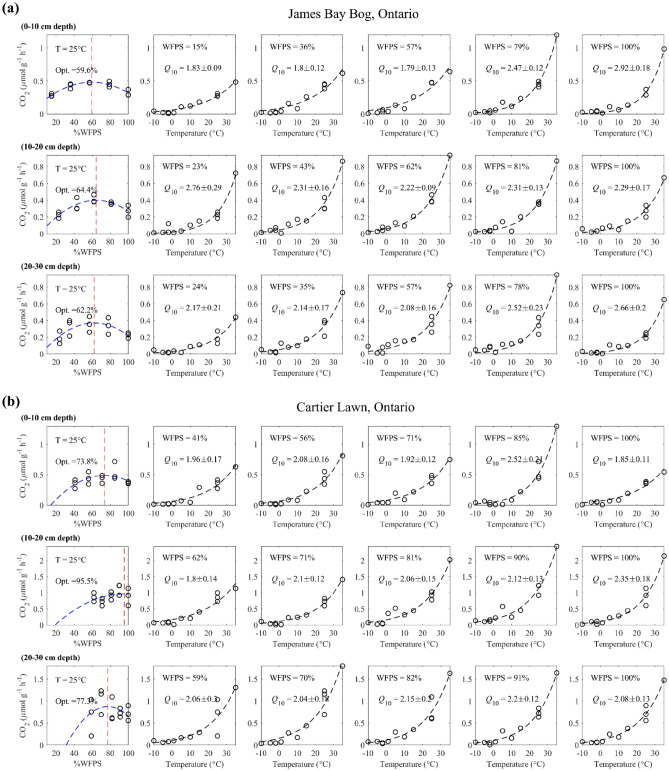
Examples of CO_2_ production rates measured for peat samples from one boreal (James Bay Bog, **a**) and one temperate site (Cartier Lawn, **b**) at different moisture contents (%WFPS; see Table 1). The rates are fitted with Eq. (1) for temperature and Eq. (5) for the moisture dependence. The effect of moisture variation at a fixed incubation temperature (25 °C) is shown in the first leftmost column; the optimum moisture levels for maximum CO_2_ production are indicated on the panels. The effects of temperature variations on the CO_2_ production at varying peat moisture contents are shown in the next five columns (in increasing order of %WFPS) with the fitted *Q*_10_ values. The results for the other sites are presented in Supplementary Figures S2 to S6.

**Figure 3:**
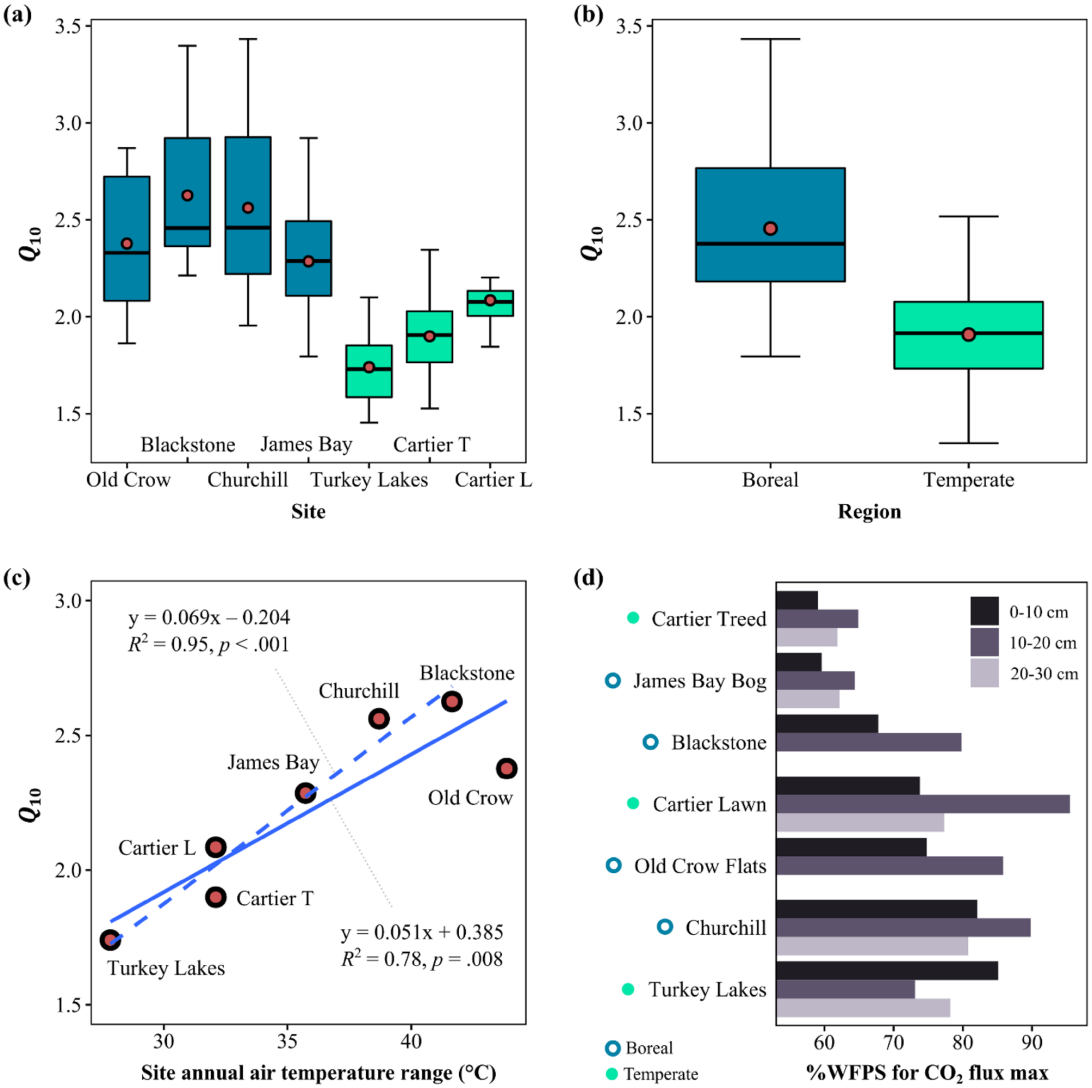
Boxplots of *Q*_10_ values calculated from incubations at different moisture contents and peat depths grouped by peatland site (**a**) and by ecoclimate zone (**b**). Boxes extend from the first to the third quartile with the inside horizontal line corresponding to the second quartile (median). Vertical lines extend to the 1.5 interquartile range of each box. The red circles (in panels a and b) indicate mean (*i.e.*, average) *Q*_10_ values. The latter are used in the linear regression analysis against the site air temperatures and the annual temperature range, *i.e.*, the difference between coldest and warmest month (panel **c**; for monthly results, see Supplementary Figure S7). Dashed fitted line in plot (**c**) represents the linear trend without the Old Crow Flats site data. The bar graphs (**d**) compare the optimum moisture levels for CO_2_ production by sampling depth and site.

For all sites, depth intervals and moisture contents, the CO_2_ production rates increased exponentially with temperature (Fig. 2 and Supplementary Figures S2-6). The *Q*_10_ distributions extracted from the exponential temperature fits to Eq. (1) are summarized in Fig. 3 for the individual sampling sites (panel a) and grouped according to ecoclimate zone (panel b). Mean *Q*_10_ values differed significantly between the seven sites [*F*(6, 88) = 14.18, *p* < 0.001] (Supplementary Table S2), although for the boreal ecoclimate zone the intra-site variance exceeded the differences in mean *Q*_10_ among the four sites [*F*(3, 46) = 2.01, *p* = 0.126]. While the differences between the Cartier Lawn (temperate) and James Bay Bog (boreal) rates were not statistically significant [*t*(21) = 2.01, *p* = 0.057], the mean James Bay Bog *Q*_10_ value was distinctly higher than that of Cartier Lawn (Fig. 3a). Thus, when the data from the sites were grouped according to ecoclimate zone (Fig. 3b), the difference between the two ecoclimate zones was significant [*t*(83) = 7.89, *p* < 0.001], with the boreal peat samples having a higher average *Q*_10_ value (mean = 2.45, SD = 0.41) than the temperate peat samples (mean = 1.91, SD = 0.25).

The mean *Q*_10_ values of the sites were inversely corelated to air temperature (for temperature records covering the 1981–2010 period). This was true whether considering monthly or yearly averaged site temperatures (Supplementary Figure S7). Thus, colder sites yielded higher mean *Q*_10_ values than warmer sites. Using simple linear regressions, the differences in the annual mean air temperature explained 74% of the observed variations in mean *Q*_10_ values [*R*^2^ = 0.74, *F*(1, 5) = 14.12, *p* = 0.013]. In addition, the site mean *Q*_10_ values correlated positively with the annual temperature range, expressed as the difference between the coldest and the warmest month [*R*^2^ = 0.78, *F*(1, 5) = 18.01, *p* = 0.008] (Fig. 3c). Note that, without the Old Crow Flats site, an even stronger linear trend emerged [*R*^2^ = 0.95, *F*(1, 4) = 82.13, *p* < 0.001].

### Moisture dependence of CO_2_ production rates

The rates in the 25 °C incubations systematically exhibited downward concave trends with respect to moisture content (%WFPS) (Fig. 2 and Supplementary Figures S2-6). The optimum moisture contents fell mostly in the range 60–90% as shown in Fig. 3d. For all the sites, except the Turkey Lakes swamp, the optimum WFPS increased between the near surface depth interval (0–10 cm) and the depth interval below (10–20 cm), although this trend did not continue when moving to the deepest depth interval (20–30 cm). The optimum WFPS in the top layer varied notably from site to site, but without a systematic differentiation between boreal and temperate sites (Fig. 3d). Overall, the variability in *Q*_10_ values was related in descending order to the variations in the following variables: ecoclimate zone > soil moisture > depth interval (Figure S8).

### CO_2_ production rates at low temperatures (−10 °C ≤ T ≤ 10 °C)

**Figure 4:**
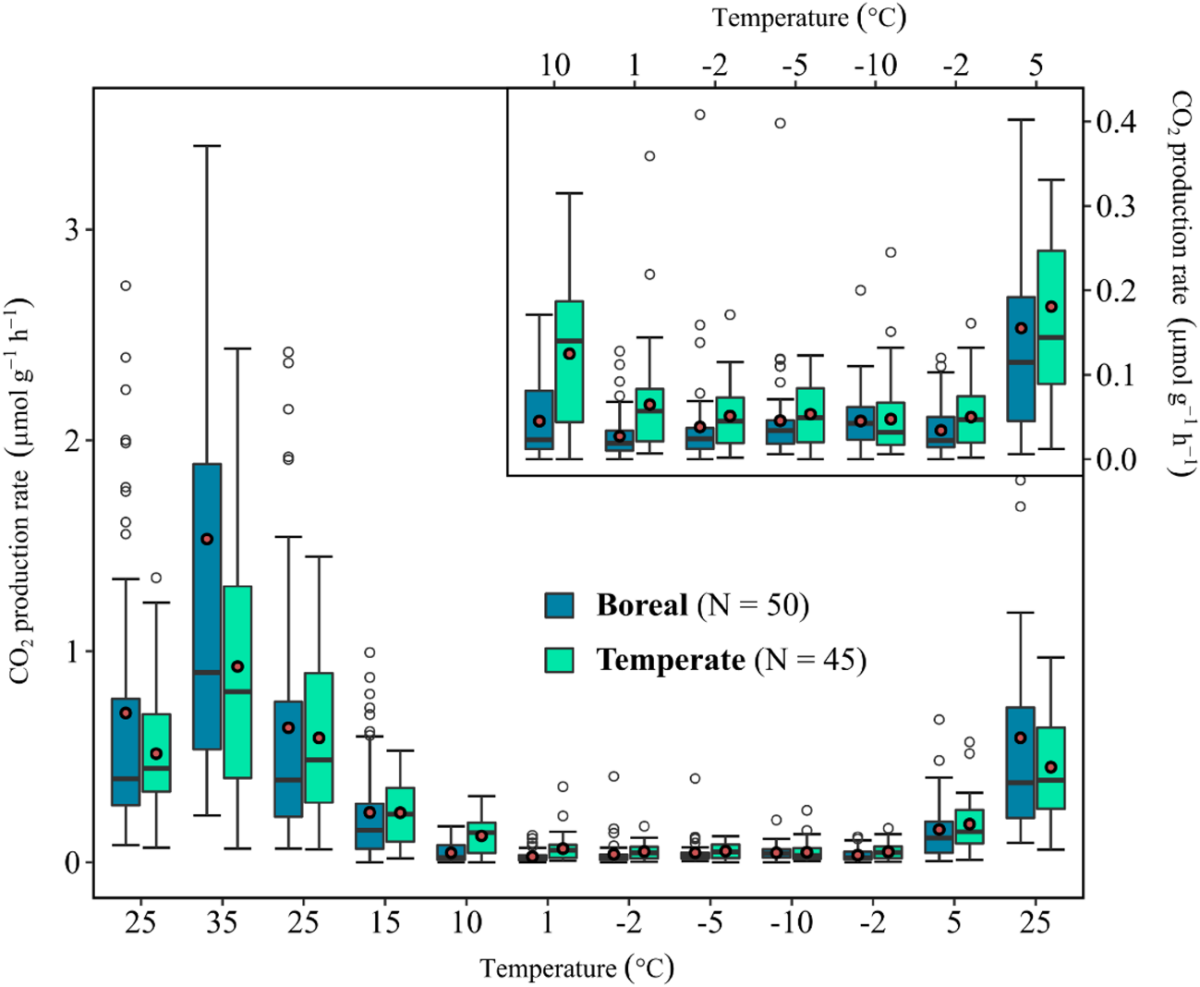
Boxplots of the peat CO_2_ production rates for the 12 incubation temperature settings grouped by ecoclimate (N represents the number of data points). The incubation temperature changed in sequential order from the first 25 °C to the last 25 °C, from left to right along the x-axis. The inset plot zooms into the results for the low temperature range (≤ 10 °C).

Because the ecoclimate site location emerged as the variable with the highest explanatory power, the CO_2_ production rate data from all sites in each of the ecoclimate zones were binned together. The two resulting ecoclimate datasets are shown in Fig. 4, arranged from left to right according to the sequence of temperature steps imposed in the incubations. For both ecoclimate zones, the CO_2_ production rates at 25 °C measured during the first and third temperature steps (that is, before the freezing event), as well as the 25 °C rates measured during the last temperature step (that is, after the freezing event) were not significantly different from one another according to a one-way ANOVA analysis of temperate [*F*(2,132) = 2.282, *p* = 0.106] and boreal [*F*(2,147) = 0.485, *p* = 0.617] rates.

The inset of Fig. 4 shows the data for the low temperature range (≤ 10 °C) that encompasses the period of freezing temperatures (i.e., the sequence of − 2, − 5, − 10 and − 2 °C steps). As can be seen, measurable CO_2_ production rates were detectable even during the sub-zero conditions. The mean rates were higher for the temperate than boreal locations, apart from the − 10 °C rates. Interestingly, the expected decreasing trend of the rates with decreasing temperature was not observed. In fact, for each ecoclimate zone, the mean rates remained relatively constant over the − 2 to − 10 °C range, with values comprised between 0.047 and 0.053 µmol g^−1^ h^−1^ for the temperate and between 0.024 and 0.040 µmol g^−1^ h^−1^ for the boreal peat samples. Furthermore, following thaw, the mean CO_2_ production rates observed at 5 °C were similar (temperate samples) or even higher (boreal samples) than those measured at 10 °C before freezing.

### CO_2_ production rates: model fits

**Figure 5:**
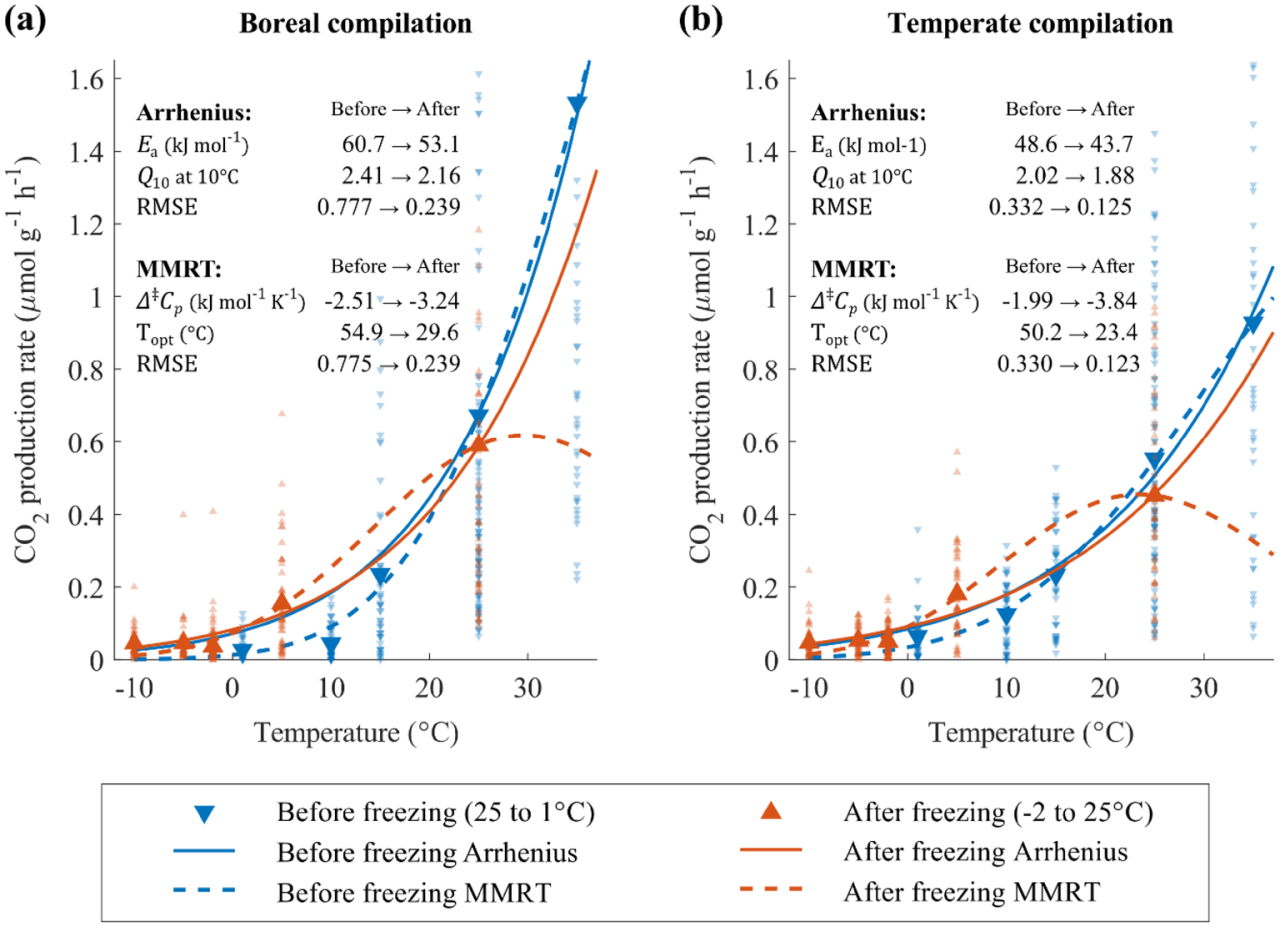
The grouped CO_2_ production rates for boreal (**a**) and temperate (**b**) ecoclimate (as in Fig. 4). The data points and fitted lines correspond to one of the two cases: before or after the start of sub-zero temperatures (*i.e.*, ‘before freezing’: from the first temperature setting at 25 °C to 1 °C; and “after freezing”: from the − 2 °C to the last 25 °C setting; see Fig. 4 x-axis for the incubation temperature steps). Data are fitted to both the Arrhenius and MMRT models (see Methods). The light symbols show all the rate data collected at a given temperature, the darker symbols are the mean rates.

The CO_2_ production rates grouped according to ecoclimate zones were fitted to both the Arrhenius equation and the MMRT models. Fits were obtained separately for rates measured before freezing (i.e., for temperatures ranging between 1 and 35 °C) and after the start of freezing (i.e., for temperatures ranging from − 10 to 25 °C). The fitted curves and the resulting model parameter values can be found on Fig. 5, together with the observed mean CO_2_ production rates. Note that the model fitting was done using all the available rate data, not just the mean rates.

For data above 10 °C, the Arrhenius fitted curves closely matched the mean CO_2_ production rates, both before and after the freezing event. For both ecoclimate groups, the Arrhenius fits yielded activation energies (*E*_a_) after freezing that were 10–12% lower than before freezing. At temperatures ≤ 10 °C, the Arrhenius equation did not reproduce the temperature trends of the mean CO_2_ rates. In particular, the Arrhenius equation did not account for the near-constant rates in the + 1 to − 10 °C range, or for the differences between rates measured before and after the onset of freezing.

The MMRT model yielded better fits to the average CO_2_ production rates measured at the lower temperatures (≤ 10 °C), but only if the before and after freezing data were fitted separately. For both ecoclimates, the fits yielded lower optimum temperatures and more negative ${\Delta }^{\ddagger }{C}_{p}$ values for the rate data collected after the onset of freezing. As a result, the shape of the fitted MMRT curves for the data collected during and after the freezing event deviated significantly from the simple exponential, or Arrhenius-type, temperature dependence.

## Discussion

### CO_2_ production kinetics: ecoclimate zones

On average, the boreal sites exhibit higher *Q*_10_ values for CO_2_ production than their temperate counterparts (Fig. 3a-b) although, within any given site, *Q*_10_ values vary considerably with changes in moisture content and peat depth (Fig. 2). The strong dependence of *Q*_10_ on the ecoclimate zone is in line with previous work that reports higher temperature sensitivities of the decomposition of soil organic matter under colder climate conditions^26–28^. Some studies have attributed observed regional *Q*_10_ variability more to soil types and land use^22,31,35^. In the present study, we attempted to limit the influence of these variables by only including soils from undisturbed peatlands.

The annual air temperature range (i.e., the difference between the coldest and the warmest month) explains most of the spread in the mean *Q*_10_ values between the seven sites (Fig. 3c). Possibly, this reflects an adaptive response of the soil heterotrophic communities to the yearly temperature range. The temperature-dependent microbial production of different extracellular enzymes can, for instance, modulate the overall temperature sensitivity of soil respiration^50^. Soil temperatures, however, may diverge significantly from the air temperature, especially in the colder, sub-arctic regions due to the insulating effects of snow and vegetation cover^52–54^. Whether this may help explain the apparent departure of the mean *Q*_10_ value for the Old Crow Flats site from the trend line in Fig. 3c remains to be seen.

Tropical peat soils have *Q*_10_ values (~ 1.3–1.8)^55^ that generally fall below the mean *Q*_10_ values observed here, thus suggesting a broad latitudinal trend that could help attenuate the response of global peatland CO_2_ emissions to ongoing and future climate warming^29^. According to climate model projections, by the end of the century most peatlands may have transitioned into a warmer climate zone^40^ (Supplementary Figure S1). If climate adaptation reduces the temperature sensitivity of peatland CO_2_ production (e.g., by lowering current *Q*_10_ values of boreal peat soils to temperate values), peat decomposition rates will still increase but the slope of the increase would be less steep than anticipated without thermal adaptation^56^. Further research is required, however, to determine how long it would take for thermal adaptation to readjust latitudinal gradients of temperature sensitivities under future climate conditions and, thus, to more realistically account for the feedback to climate of peat soil carbon cycling^57–59^.

A complete mechanistic understanding of the thermal adaptation of soil microbes, and hence CO_2_ production kinetics, remains elusive^32,60,61^. Nonetheless, empirical model parameter values obtained under relevant environmental conditions can still significantly improve regionalized models of carbon emissions from peatlands^56,62^. For example, the most recent version of the Canadian peatland model (CaMP v2.0) incorporates variable SOC decay rate coefficients for different peatland ecological types but does not explicitly consider differences due to ecoclimate^17^. Our results show that the temperature sensitivity of CO_2_ production may vary significantly and, possibly, systematically, depending on ecoclimate location. This finding therefore implies that including ecoclimate-specific temperature sensitivities of peat mineralization in models such as CaMP could significantly reduce parameter uncertainties when assessing the contribution of peat decomposition to continental-scale CO_2_ budgets.

### CO_2_ production kinetics: soil moisture

The effect of soil moisture was evaluated using the CO_2_ production rates at 25 °C for which we have the most complete set of replicate measurements. The concave down shapes of the moisture response curves (Fig. 2) are consistent with other studies. However, the optimum WFPS values obtained here for the peat soils (Fig. 3d) tend to be higher than the ~ 60% moisture levels typically observed for more upland and mineral soils (e.g., ^37,45,63–66^).

Optimum moisture levels for soil respiration emerge from complex interactions among physiochemical (e.g., pore water distribution, oxygen availability, and solute diffusion^67,68^) and biological factors (e.g., microbial enzyme activity regulation^51^). The general shift to higher optimum moisture levels in peat soils is likely, at least in part, due to their high organic matter content and complex (multi-domain) pore structure compared to non-peat soils^36,69,70^. Still, the organic carbon concentrations of our peat samples fall in a fairly narrow range (Table 1), while the optimum WFPS levels at 25 °C vary between 59 and 96% (Fig. 2 and Supplementary Figures S2-6).

The variations in the optimum WFPS for CO_2_ production appear to be related to in situ water table conditions, as inferred from the peatland type and microtopography at the sampling location (Table 1). For example, the peat soils from the Blackstone and Cartier Treed sites were collected on hummocks, where the water table is relatively deep below the surface and the top peat often drier than in sunken areas of the landscape, such as hollows^71^. Similarly, prior to our sampling, the James Bay Bog site had been experiencing a lowering of the water table due to nearby mining operations^72^. By contrast, the soils in the permafrost tundra with fen type vegetation (e.g., *Eriophorum*) covers are characterized by shallow water tables and wet conditions^73^. While speculative, the correlation between the water table depth and the measured optimum WFPS may reflect an adaptation of peatland microbial communities to the prevailing in situ moisture conditions^10,59^.

Rewetting of peatlands has been proposed as a management strategy to restore their carbon sink function (e.g., ^74,75^). The experimental results presented here support a systematic decline of the CO_2_ production rates at full saturation (i.e., 100% WFPS). However, the complete evaluation of the effect of peatland rewetting on carbon emissions must consider the concomitant increases in CH_4_ release due to the decreased availability of oxygen^76^. Our results further indicate that CO_2_ production rates also decrease when the moisture level drops below the optimum WFPS. In the field, drier conditions due to a lowering of the water table would shift the zone of optimum moisture level downwards and, therefore, not necessarily translate in a reduction of the CO_2_ emissions at the soil surface.

In addition, there is evidence that, given enough time, the microbial decomposer communities may adapt to drier conditions. Arnold et al.^67^ for example, showed that in long-term (> 300 days) incubations the simulated drainage of wetland soils resulted in cumulatively more organic carbon being mineralized. Recent work has also been exploring the role of moisture on the temperature sensitivity of soil CO_2_ production (e.g., ^37,69^). Together, the results of these studies call for more systematic and data-driven approaches to represent the regulating role of soil moisture in soil carbon process models^67,68,77^ that, in turn, would improve the reliability of predicted responses of peatland carbon budgets to changes in soil moisture regime due to climate and land use changes.

### CO_2_ production kinetics: impact of freezing

The production of CO_2_ at sub-zero temperatures in both ecoclimate groups exhibits small but measurable rates through the − 2 to − 10 °C temperature range (Fig. 4). The rates measured here are similar to sub-zero rates in the same temperature range reported in other low-temperature incubation experiments (see, for example, the data compiled by Natali et al.^8^). Most importantly, the observed trends do not follow the exponential drop of the rates with cooling below 0 °C that is often assumed^8^. On average, the sub-zero CO_2_ production rates are remarkably constant and of comparable magnitude for both ecoclimates (Fig. 5). The lack of a temperature dependence of the average sub-zero rates is in apparent contradiction with standard reaction rate models.

The higher-than-expected CO_2_ production rates at temperatures below 0 °C could reflect an enhanced release of biodegradable organic substrates caused by the freezing of the soil that, in the case of peat soils, may be facilitated by the high moisture contents^34,78–81^. Such a release of labile organic substrates is supported by the higher rates measured at 5 °C after thawing, compared to the rates measured at 10 °C prior to freezing. Thus, a possible explanation for the near-constant rates in the − 2 to − 10 °C range is that the enhanced supply of labile organic compounds counterbalances the lowering of temperature below 0 °C. Because more labile compounds have lower activation energies of degradation, they also result in lower temperature sensitivities. This is consistent with the lower fitted *E*_*a*_ and *Q*_*10*_ values after the freezing event for both ecoclimate groups (the values are given on Fig. 5).

The divergence of the temperature trends of the CO_2_ production rates before and after freezing is not captured by the Arrhenius rate equation, even after adjusting the *E*_*a*_ values (Fig. 5). By contrast, the MMRT model is able to account for the enhanced rates observed after thawing in the 0 to 10 °C range. For both ecoclimate groups, the post-thaw MMRT fits yield more negative ${\Delta }^{\ddagger }{C}_{p}$ values and a lowering of the optimum temperatures (*T*_*opt*_). These changes are in line with the “Enzyme Rigidity Hypothesis” (see Alster et al.^38^ for details), according to which cold-adapted enzymes are more sensitive, or “less rigid,” than enzymes adapted to warmer temperatures. Thus, in addition to releasing more labile organic substrates, the cooling to sub-zero temperatures could also have been accompanied by the selective activation of “cold-adapted enzymes” by the microbial communities.

When dynamic enzyme responses modulate the rates of organic matter degradation, the temperature dependence is expected to depart from the Arrhenius equation and exhibit an optimum biological temperature (*T*_*opt*_)^37^. However, the predicted post-freezing *T*_*opt*_ values, 23.4 and 29.6 °C, would need to be verified by Increasing the experimental temperature range well above 25 °C (which was the final temperature setting in our experiments), and including more frequent measurement intervals^38,49^. Nonetheless, the MMRT provides a more flexible approach that could be useful in modeling the in-field observed seasonal hysteresis in the temperature trends of CO_2_ emissions (e.g., ^20,30,58^), as well as CH_4_ emissions^82–84^.

### Implications for CO_2_ emissions during the NGS

The failure of the Arrhenius and MMRT models to reproduce the observed temperature trends of the sub-zero CO_2_ production rates is not entirely surprising. Both models only account for the temperature dependence of molecular reaction rates. In addition to increasing the supply of labile substrates, freezing may have other consequences for the cryogenic soil environment, including changes in the distribution of unfrozen water and the transport pathways of solutes and gases^33,64,70,85^. Together, these changes modulate the measured rates at which CO_2_ is released from the soil. Thus, it may be advisable not to extend the use of reaction rate models to simulate sub-zero CO_2_ production rates in soils, or those portions of the soils, that undergo seasonal freezing. To properly represent frozen peat soils, future experimental work should consider measuring CO_2_ production over longer time spans than in the present study, weeks to months rather than the 48 h per temperature step here, and extend the temperature range covered.

The experimental results imply that freezing impacts the rates of CO_2_ production upon thawing; this is especially evident from the 5 °C post-thawing data (Figs. 4 and 5). Typically, the increase in CO_2_ emission fluxes during spring thaw observed in the field are attributed to a combination of the pulsed release of trapped gas that accumulated under the snow and ice layer during winter^86^ and de novo microbial CO_2_ production due to the sudden increase in temperature and oxygen supply upon snowmelt^4^. Our results suggest that the enhanced access to relatively labile organic compounds mobilized during freezing may additionally contribute to the observed post-thaw CO_2_ pulses.

It will be important to further determine whether the post-thaw CO_2_ rate enhancement observed in our experiments is short-lived, and possibly insignificant in the overall NGS CO_2_ emission budget, or whether it could help sustain CO_2_ production throughout the shoulder season^30^. The importance of enhanced carbon loss from peatlands induced by encroaching permafrost thaw is now well recognized^87^. However, the role of shallow freeze–thaw cycles on NGS CO_2_ emissions need to be better quantified and the responsible mechanisms understood. At higher latitudes, freeze–thaw cycles are expected become more frequent in the springtime, because trends in snow cover (and its temperature insulating effect) are decreasing^20,88^.

## Summary and conclusions

This study highlights the statistically significant variations in the temperature sensitivity of peat soil CO_2_ production rates between the cold-temperate and boreal ecoclimate zones. Given the higher *Q*_10_ values, in combination with spatial and seasonal patterns of global warming, boreal peatlands may increase future NGS CO_2_ losses to a larger degree than temperate peatlands. The variable temperature sensitivities under different climate conditions need to be accounted for when assessing future global trajectories of peatland carbon pool stability.

Peatland soils tend to support high organic matter mineralization activity at higher moisture levels compared to well-drained mineral soils. Our findings suggest that the optimum moisture level for maximum CO_2_ production is a function of peatland type (e.g., bog or fen) and microtopography (e.g., samples from hummock or hollow), which, in turn, are related to the relative position of the water table. The variations in optimum moisture content may thus reflect an adaptation of the resident microbial communities to the prevailing moisture conditions.

Freezing of the peat samples causes a shift in the temperature response curve for the CO_2_ production rates. This shift is captured by the MMRT model, but not by the conventional Arrhenius equation. Furthermore, the reaction rate models are unable to account for the near-constant CO_2_ production rates measured across the sub-zero temperature range (− 2 to − 10 °C). More research is needed to unravel the various processes and structural properties controlling CO_2_ production in frozen peat soils and their response to the changes in temperature and hydrology under climate warming. Only with this improved mechanistic understanding supported by precise rate data will process models be able to grapple with the implications of climate and land use changes for NGS carbon budgets of northern peatlands.

## Supplementary Information

Supplementary Information.

## References

[1] Huang J, Zhang X, Zhang Q, Lin Y, Hao M, Luo Y (2017). Recently amplified arctic warming has contributed to a continual global warming trend. Nat. Clim. Chang..

[2] Zhang X, Flato G, Kirchmeier-Young M, Vincent LA, Wan H, Wang X, Bush E, Lemmen DS (2019). Changes in temperature and precipitation across Canada. Canada’s Changing Climate Report.

[3] Koenigk T, Brodeau L, Graversen RG, Karlsson J, Svensson G, Tjernström M (2013). Arctic climate change in 21st century CMIP5 simulations with EC-Earth. Clim. Dyn..

[4] Arndt KA, Lipson DA, Hashemi J, Oechel WC, Zona D (2020). Snow melt stimulates ecosystem respiration in Arctic ecosystems. Glob. Change Biol..

[5] Commane R, Lindaas J, Benmergui J, Luus KA, Chang RYW, Daube BC (2017). Carbon dioxide sources from Alaska driven by increasing early winter respiration from Arctic tundra. Proc. Natl. Acad. Sci. U.S.A..

[6] Euskirchen ES, Bret-Harte MS, Shaver GR, Edgar CW, Romanovsky VE (2017). Long-term release of carbon dioxide from Arctic Tundra Ecosystems in Alaska. Ecosystems.

[7] Webb EE, Schuur EAG, Natali SM, Oken KL, Bracho R, Krapek JP (2016). Increased wintertime CO2 loss as a result of sustained tundra warming. J. Geophys. Res. Biogeosci..

[8] Natali SM, Watts JD, Rogers BM, Potter S, Ludwig SM, Selbmann AK (2019). Large loss of CO2 in winter observed across the northern permafrost region. Nat. Clim. Chang..

[9] Rafat A, Rezanezhad R, Quinton WL, Humphreys ER, Webster K, Van Cappellen P (2021). Non-growing season carbon emissions in a northern peatland are projected to increase under global warming. Nature Communications Earth & Enviornment.

[10] Yarwood SA (2018). The role of wetland microorganisms in plant-litter decomposition and soil organic matter formation: A critical review. FEMS Microbiol. Ecol..

[11] Yu ZC (2012). Northern peatland carbon stocks and dynamics: A review. Biogeosciences.

[12] Keenan TF, Williams CA (2018). The terrestrial carbon sink. Annu. Rev. Environ. Resour..

[13] Stocker BD, Yu Z, Massa C, Joos F (2017). Holocene peatland and ice-core data constraints on the timing and magnitude of CO2 emissions from past land use. Proc. Natl. Acad. Sci..

[14] Webster KL, Bhatti JS, Thompson DK, Nelson SA, Shaw CH, Bona KA (2018). Spatially-integrated estimates of net ecosystem exchange and methane fluxes from Canadian peatlands. Carbon Balance Manage..

[15] Byun E, Finkelstein SA, Cowling SA, Badiou P (2018). Potential carbon loss associated with post-settlement wetland conversion in southern Ontario, Canada. Carbon Balance Manag.

[16] Lei J, Guo X, Zeng Y, Zhou J, Gao Q, Yang Y (2021). Temporal changes in global soil respiration since 1987. Nat. Commun..

[17] Bona KA, Shaw C, Thompson DK, Hararuk O, Webster K, Zhang G (2020). The Canadian model for peatlands (CaMP): A peatland carbon model for national greenhouse gas reporting. Ecol. Model..

[18] Brooks PD, McKnight D, Elder K (2005). Carbon limitation of soil respiration under winter snowpacks: Potential feedbacks between growing season and winter carbon fluxes. Glob. Change Biol..

[19] Helbig M, Chasmer LE, Desai AR, Kljun N, Quinton WL, Sonnentag O (2017). Direct and indirect climate change effects on carbon dioxide fluxes in a thawing boreal forest–wetland landscape. Glob. Change Biol..

[20] Zhang T, Wang G, Yang Y, Mao T, Chen X (2015). Non-growing season soil CO2 flux and its contribution to annual soil CO2 emissions in two typical grasslands in the permafrost region of the Qinghai-Tibet Plateau. Eur. J. Soil Biol..

[21] Grosse G, Harden J, Turetsky M, McGuire AD, Camill P, Tarnocai C (2011). Vulnerability of high-latitude soil organic carbon in North America to disturbance. J. Geophys. Res..

[22] Hamdi S, Moyano F, Sall S, Bernoux M, Chevallier T (2013). Synthesis analysis of the temperature sensitivity of soil respiration from laboratory studies in relation to incubation methods and soil conditions. Soil Biol. Biochem..

[23] Conant RT, Ryan MG, Ågren GI, Birge HE, Davidson EA, Eliasson PE (2011). Temperature and soil organic matter decomposition rates—synthesis of current knowledge and a way forward. Glob. Change Biol..

[24] Davidson EA, Janssens IA (2006). Temperature sensitivity of soil carbon decomposition and feedbacks to climate change. Nature.

[25] Fang C, Smith P, Moncrieff JB, Smith JU (2005). Similar response of labile and resistant soil organic matter pools to changes in temperature. Nature.

[26] Koven CD, Hugelius G, Lawrence DM, Wieder WR (2017). Higher climatological temperature sensitivity of soil carbon in cold than warm climates. Nat. Clim. Chang..

[27] Li J, Nie M, Pendall E, Reich PB, Pei J, Noh NJ (2020). Biogeographic variation in temperature sensitivity of decomposition in forest soils. Glob. Change Biol..

[28] Li J, Pei J, Pendall E, Fang C, Nie M (2020). Spatial heterogeneity of temperature sensitivity of soil respiration: A global analysis of field observations. Soil Biol. Biochem..

[29] Niu B, Zhang X, Piao S, Janssens IA, Fu G, He Y (2021). Warming homogenizes apparent temperature sensitivity of ecosystem respiration. Sci. Adv..

[30] Wang J, Wu Q, Yuan Z, Kang H (2020). Soil respiration of alpine meadow is controlled by freeze-Thaw processes of active layer in the permafrost region of the Qinghai-Tibet Plateau. Cryosphere.

[31] Wang Q, Zhao X, Chen L, Yang Q, Chen S, Zhang W (2019). Global synthesis of temperature sensitivity of soil organic carbon decomposition: Latitudinal patterns and mechanisms. Funct. Ecol..

[32] Bradford MA, Wieder WR, Bonan GB, Fierer N, Raymond PA, Crowther TW (2016). Managing uncertainty in soil carbon feedbacks to climate change. Nat. Clim. Chang..

[33] Pi K, Bieroza M, Brouchkov A, Chen W, Dufour LJP, Gongalsky KB (2021). The cold region critical zone in transition: Responses to climate warming and land use change. Annu. Rev. Environ. Resour..

[34] Fuss CB, Driscoll CT, Groffman PM, Campbell JL, Christenson LM, Fahey TJ (2016). Nitrate and dissolved organic carbon mobilization in response to soil freezing variability. Biogeochemistry.

[35] Meyer N, Welp G, Amelung W (2018). The temperature sensitivity (Q10) of soil respiration: Controlling factors and spatial prediction at regional scale based on environmental soil classes. Global Biogeochem. Cycles.

[36] Moyano FE, Vasilyeva N, Bouckaert L, Cook F, Craine J, Curiel Yuste J (2012). The moisture response of soil heterotrophic respiration: Interaction with soil properties. Biogeosciences.

[37] Schipper LA, Petrie OJ, O’Neill TA, Mudge PL, Liáng LL, Robinson JM, Arcus VL (2019). Shifts in temperature response of soil respiration between adjacent irrigated and non-irrigated grazed pastures. Agr. Ecosyst. Environ..

[38] Alster CJ, von Fischer JC, Allison SD, Treseder KK (2020). Embracing a new paradigm for temperature sensitivity of soil microbes. Glob. Change Biol..

[39] Baldwin, K. *et al.* Vegetation Zones of Canada: a Biogeoclimatic Perspective. Sault Ste. Marie, ON, Canada: Natural Resources Canada, Canadian Forest Service. Great Lake Forestry Center. https://open.canada.ca/data/en/dataset/22b0166b-9db3-46b7-9baf-6584a3acc7b1 (2019).

[40] Beck HE, Zimmermann NE, McVicar TR, Vergopolan N, Berg A, Wood EF (2018). Present and future köppen-geiger climate classification maps at 1-km resolution. Sci. Data.

[41] Gardner WH, Klute A (1986). Water content. Methods of soil analysis: Physical and mineralogical methods, agronomy series 9 (Part 1).

[42] Webster KL, Creed IF, Bourbonnière RA, Beall FD (2008). Controls on the heterogeneity of soil respiration in a tolerant hardwood forest. J. Geophys. Res..

[43] Quinton WL, Baltzer JL (2013). The active-layer hydrology of a peat plateau with thawing permafrost (Scotty Creek, Canada). Hydrogeol. J..

[44] Davidson EA, Savage K, Verchot LV, Navarro R (2002). Minimizing artifacts and biases in chamber-based measurements of soil respiration. Agric. For. Meteorol..

[45] Rezanezhad F, Couture RM, Kovac R, O’Connell D, Van Cappellen P (2014). Water table fluctuations and soil biogeochemistry: An experimental approach using an automated soil column system. J. Hydrol..

[46] Fang C, Moncrieff JB (2001). The dependence of soil CO2 efflux on temperature. Soil Biol. Biochem..

[47] Alster CJ, Koyama A, Johnson NG, Wallenstein MD, von Fischer JC (2016). Temperature sensitivity of soil microbial communities: An application of macromolecular rate theory to microbial respiration. J. Geophys. Res. Biogeosci..

[48] Hobbs JK, Jiao W, Easter AD, Parker EJ, Schipper LA, Arcus VL (2013). Change in heat capacity for enzyme catalysis determines temperature dependence of enzyme catalyzed rates. ACS Chem. Biol..

[49] Robinson JM, O’Neill TA, Ryburn J, Liang LL, Arcus VL, Schipper LA (2017). Rapid laboratory measurement of the temperature dependence of soil respiration and application to changes in three diverse soils through the year. Biogeochemistry.

[50] Schipper LA, Hobbs JK, Rutledge S, Arcus VL (2014). Thermodynamic theory explains the temperature optima of soil microbial processes and high Q10 values at low temperatures. Glob. Change Biol..

[51] Webster KL, Creed IF, Malakoff T, Delaney K (2014). Potential Vulnerability of Deep Carbon Deposits of Forested Swamps to Drought. Soil Sci. Soc. Am. J..

[52] Loranty MM, Abbott BW, Blok D, Douglas TA, Epstein HE, Forbes BC (2018). Reviews and syntheses: Changing ecosystem influences on soil thermal regimes in northern high-latitude permafrost regions. Biogeosciences.

[53] Roy-Léveillée P, Burn CR, Mcdonald ID (2014). Vegetation-Permafrost Relations within the Forest-Tundra Ecotone near Old Crow, Northern Yukon, Canada. Permafr. and Periglac. Process..

[54] Zhang Y, Sherstiukov AB, Qian B, Kokelj SV, Lantz TC (2018). Impacts of snow on soil temperature observed across the circumpolar north. Environ. Res. Lett..

[55] Sjögersten S, Aplin P, Gauci V, Peacock M, Siegenthaler A, Turner BL (2018). Temperature response of ex-situ greenhouse gas emissions from tropical peatlands: Interactions between forest type and peat moisture conditions. Geoderma.

[56] Bradford MA, McCulley RL, Crowther TW, Oldfield EE, Wood SA, Fierer N (2019). Cross-biome patterns in soil microbial respiration predictable from evolutionary theory on thermal adaptation. Nature Ecology and Evolution.

[57] Frey SD, Lee J, Melillo JM, Six J (2013). The temperature response of soil microbial efficiency and its feedback to climate. Nat. Clim. Chang..

[58] Moinet GYK, Moinet M, Hunt JE, Rumpel C, Chabbi A, Millard P (2020). Temperature sensitivity of decomposition decreases with increasing soil organic matter stability. Sci. Total Environ..

[59] Naylor D, Sadler N, Bhattacharjee A, Graham EB, Anderton CR, McClure R (2020). Soil microbiomes under climate change and implications for carbon cycling. Annu. Rev. Environ. Resour..

[60] Bradford MA (2013). Thermal adaptation of decomposer communities in warming soils. Front. Microbiol..

[61] Jackson RB, Lajtha K, Crow SE, Hugelius G, Kramer MG, Piñeiro G (2017). The ecology of soil carbon: pools, vulnerabilities, and biotic and abiotic controls. Annu. Rev. Ecol. Evol. Syst..

[62] Hararuk O, Shaw C, Kurz WA (2017). Constraining the organic matter decay parameters in the CBM-CFS3 using Canadian National Forest Inventory data and a Bayesian inversion technique. Ecol. Model..

[63] Franzluebbers AJ (1999). Microbial activity in response to water-filled pore space of variably eroded southern Piedmont soils. Appl. Soil. Ecol..

[64] Rezanezhad F, Price JS, Quinton WL, Lennartz B, Milojevic T, Van Cappellen P (2016). Structure of peat soils and implications for water storage, flow and solute transport: A review update for geochemists. Chem. Geol..

[65] Stirling E, Fitzpatrick RW, Mosley LM (2020). Drought effects on wet soils in inland wetlands and peatlands. Earth Sci. Rev..

[66] Wickland KP, Neff JC (2008). Decomposition of soil organic matter from boreal black spruce forest: Environmental and chemical controls. Biogeochemistry.

[67] Arnold C, Ghezzehei TA, Berhe AA (2015). Decomposition of distinct organic matter pools is regulated by moisture status in structured wetland soils. Soil Biol. Biochem..

[68] Moyano FE, Manzoni S, Chenu C (2013). Responses of soil heterotrophic respiration to moisture availability: An exploration of processes and models. Soil Biol. Biochem..

[69] Sierra CA, Malghani S, Loescher HW (2017). Interactions among temperature, moisture, and oxygen concentrations in controlling decomposition rates in a boreal forest soil. Biogeosciences.

[70] McCarter CPR, Rezanezhad F, Quinton WL, Gharedaghloo B, Lennartz B, Price J (2020). Pore-scale controls on hydrological and geochemical processes in peat: Implications on interacting processes. Earth Sci. Rev..

[71] Strack M, Waddington JM, Bourbonniere RA, Buckton EL, Shaw K, Whittington P, Price JS (2008). Effect of water table drawdown on peatland dissolved organic carbon export and dynamics. Hydrol. Process..

[72] Leclair M, Whittington P, Price J (2015). Hydrological functions of a mine-impacted and natural peatland-dominated watershed, James Bay Lowland. J. Hydrol. Reg. Stud..

[73] Treat CC, Jones MC, Camill P, Gallego-Sala A, Garneau M, Harden JW (2016). Effects of permafrost aggradation on peat properties as determined from a pan-Arctic synthesis of plant macrofossils. J. Geophys. Res. Biogeosci..

[74] Günther A, Barthelmes A, Huth V, Joosten H, Jurasinski G, Koebsch F, Couwenberg J (2020). Prompt rewetting of drained peatlands reduces climate warming despite methane emissions. Nat. Commun..

[75] Schädel C, Bader MKF, Schuur EAG, Biasi C, Bracho R, Čapek P (2016). Potential carbon emissions dominated by carbon dioxide from thawed permafrost soils. Nat. Clim. Chang..

[76] Hemes KS, Chamberlain SD, Eichelmann E, Knox SH, Baldocchi DD (2018). A biogeochemical compromise: The high methane cost of sequestering carbon in restored wetlands. Geophys. Res. Lett..

[77] Davidson EA, Samanta S, Caramori SS, Savage K (2012). The Dual Arrhenius and Michaelis-Menten kinetics model for decomposition of soil organic matter at hourly to seasonal time scales. Glob. Change Biol..

[78] Matzner E, Borken W (2008). Do freeze-thaw events enhance C and N losses from soils of different ecosystems? A review. Eur. J. Soil Sci..

[79] Song Y, Zou Y, Wang G, Yu X (2017). Altered soil carbon and nitrogen cycles due to the freeze-thaw effect: A meta-analysis. Soil Biol. Biochem..

[80] Wang J, Song C, Hou A, Miao Y, Yang G, Zhang J (2014). Effects of freezing-thawing cycle on peatland active organic carbon fractions and enzyme activities in the Da Xing’anling Mountains. Northeast China. Environmental Earth Sciences.

[81] Wu H, Xu X, Cheng W, Fu P, Li F (2017). Antecedent soil moisture prior to freezing can affect quantity, composition and stability of soil dissolved organic matter during thaw. Sci. Rep..

[82] Bao T, Xu X, Jia G, Billesbach DP, Sullivan RC (2021). Much stronger tundra methane emissions during autumn freeze than spring thaw. Glob. Change Biol..

[83] Chang KY, Riley WJ, Crill PM, Grant RF, Saleska SR (2020). Hysteretic temperature sensitivity of wetland CH4 fluxes explained by substrate availability and microbial activity. Biogeosciences.

[84] Neumann RB, Moorberg CJ, Lundquist JD, Turner JC, Waldrop MP, McFarland JW (2019). Warming Effects of Spring Rainfall Increase Methane Emissions From Thawing Permafrost. Geophys. Res. Lett..

[85] Rezanezhad F, Price JS, Craig JR (2012). The effects of dual porosity on transport and retardation in peat: A laboratory experiment. Can. J. Soil Sci..

[86] Raz-Yaseef N, Torn MS, Wu Y, Billesbach DP, Liljedahl AK, Kneafsey TJ (2017). Large CO2 and CH4 emissions from polygonal tundra during spring thaw in northern Alaska. Geophys. Res. Lett..

[87] Waldrop MP, McFarland J, Manies K, Leewis MC, Blazewicz SJ, Jones MC (2021). Carbon fluxes and microbial activities from boreal peatlands experiencing permafrost thaw. J. Geophys. Res. Biogeosci..

[88] Pulliainen J, Luojus K, Derksen C, Mudryk L, Lemmetyinen J, Salminen M (2020). Patterns and trends of Northern Hemisphere snow mass from 1980 to 2018. Nature.

