# Supplementary material for: Temperature, moisture and freeze–thaw controls on CO2 production in soil incubations from northern peatlands
Source: Sci Rep. 2021 Dec 1;11:23219. doi: 10.1038/s41598-021-02606-3 (PMC8636591; doi:10.1038/s41598-021-02606-3)
Supplement: Supplementary file 1 — Supplementary Information. [file 41598_2021_2606_MOESM1_ESM.docx]

**Supplementary Information**

Temperature, moisture and freeze-thaw controls on CO_2_ production in soil incubations from northern peatlands

Eunji Byun^1^*, Fereidoun Rezanezhad^1^*, Linden Fairbairn^1,2^, Stephanie Slowinski^1^, Nathan Basiliko^3^, Jonathan S. Price^4^, William L. Quinton^5^, Pascale Roy-Léveillée^2,6^, Kara Webster^7^, and Philippe Van Cappellen^1^*

^1^Ecohydrology Research Group, Department of Earth and Environmental Sciences and Water Institute, University of Waterloo, ON, Canada

^2^Environment and Climate Change Canada, Canada

^3^Department of Biology and Vale Living with Lakes Centre, Laurentian University, Sudbury, ON, Canada

^4^Department of Geography and Environmental Management, University of Waterloo, Waterloo, ON, Canada

^5^Cold Regions Research Centre, Wilfrid Laurier University, Waterloo, ON, Canada

^6^Université Laval, Québec, QC, Canada

^7^Canadian Forest Service Great Lakes Forestry Centre - Natural Resources Canada, Sault Ste Marie, ON, Canada

*Corresponding authors: Eunji Byun (ebyun@uwaterloo.ca), Fereidoun Rezanezhad (frezanezhad@uwaterloo.ca), and Philippe Van Cappellen ([pvc@uwaterloo.ca](mailto:pvc@uwaterloo.ca))

Ecohydrology Research Group, Department of Earth and Environmental Sciences and Water Institute, University of Waterloo, 200 University Avenue West, Waterloo, Canada N2L 3G1


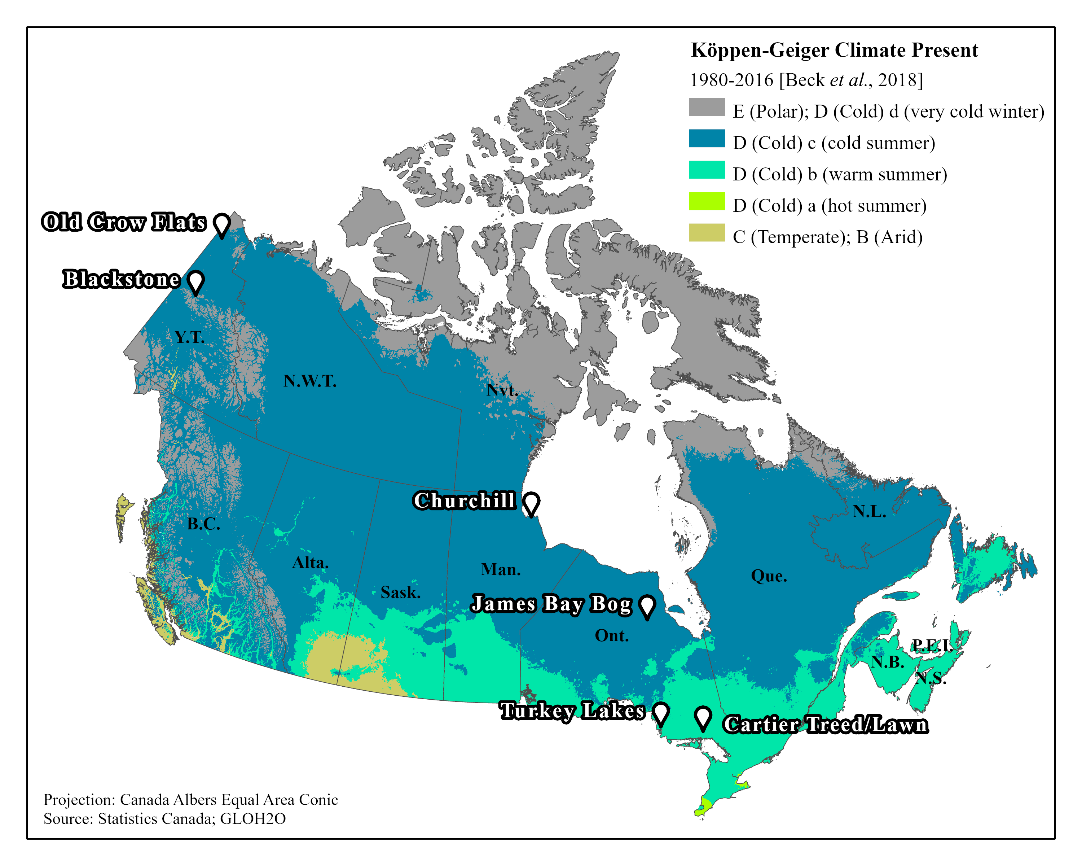


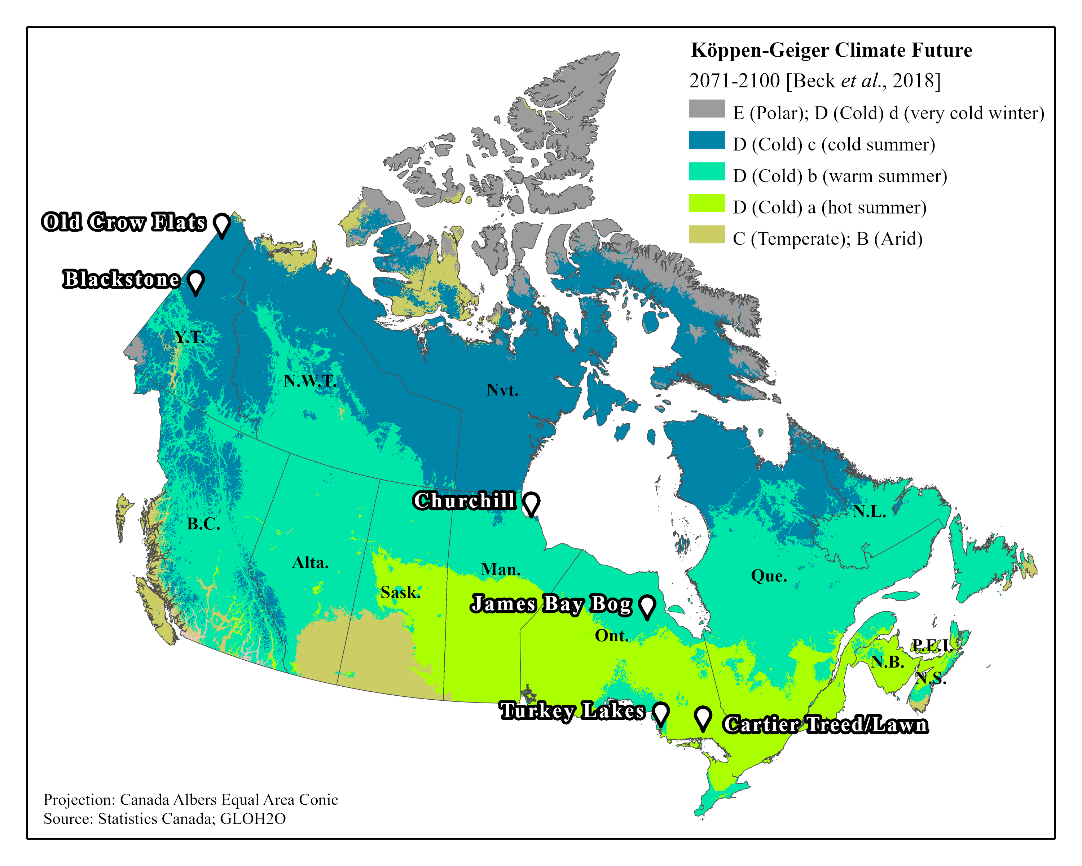


**Figure S1:** The locations of peatland sites across different climate classes in Canada according to Köppen-Geiger climate classification maps (Beck *et al.*, 2018). The top panel shows the present climate (1980-2016) and the bottom future projection based on RCP8.5 scenario. The site names are corresponding to the information in Table 1 and Figure 1 in the main text. The map was created using ArcGIS Pro (Esri).


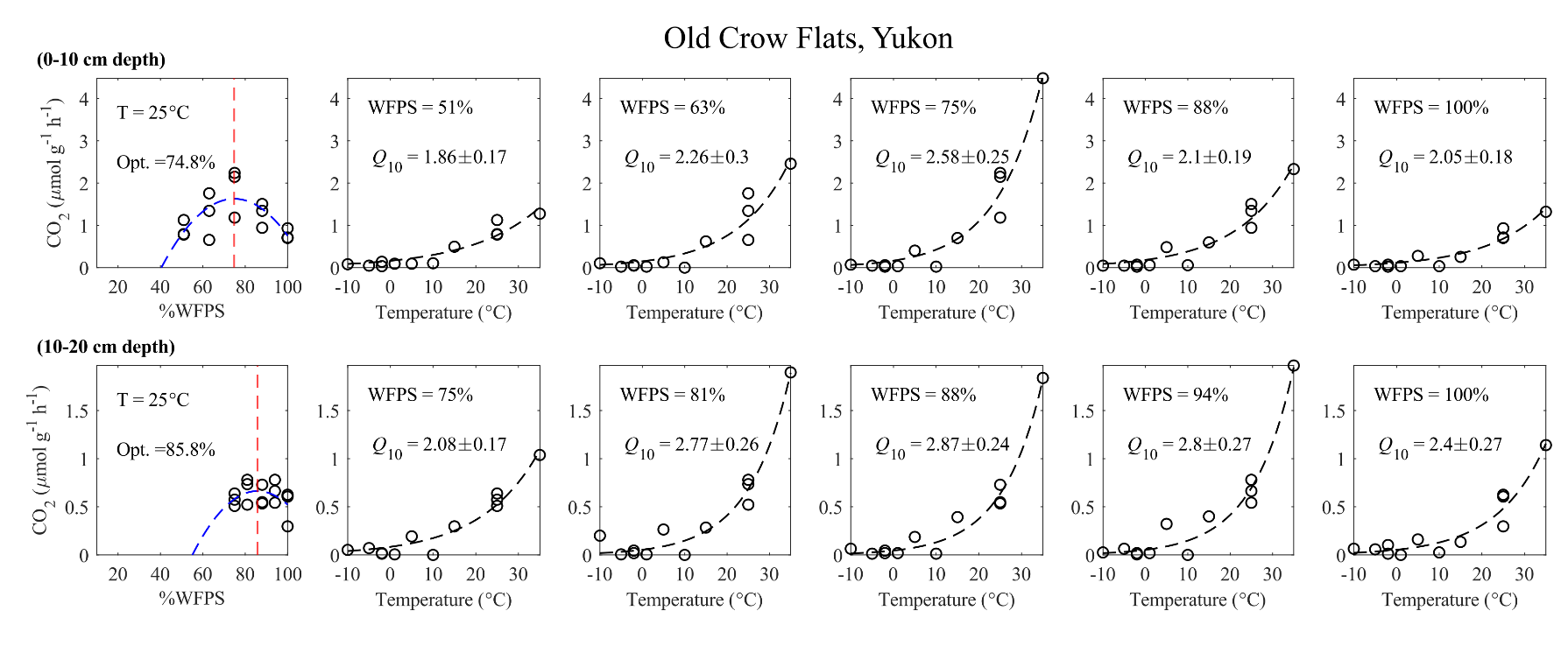


**Figure S2:** CO_2_ production rates measured in peat samples from Old Crow Flats (see Table 1 for the site information) at different moisture contents (%WFPS) and fitted with Equation (1) and Equation (5) in the main text. The effect of moisture variation at a fixed temperature (25°C) incubation is shown in the first leftmost column with the concave down trend and an optimum moisture level for the maximum CO_2_ production rate. The effects of temperature variations on CO_2_ production at varying peat moisture contents are shown in the next five columns (in increasing order of %WFPS) to derive *Q*_10_ values.


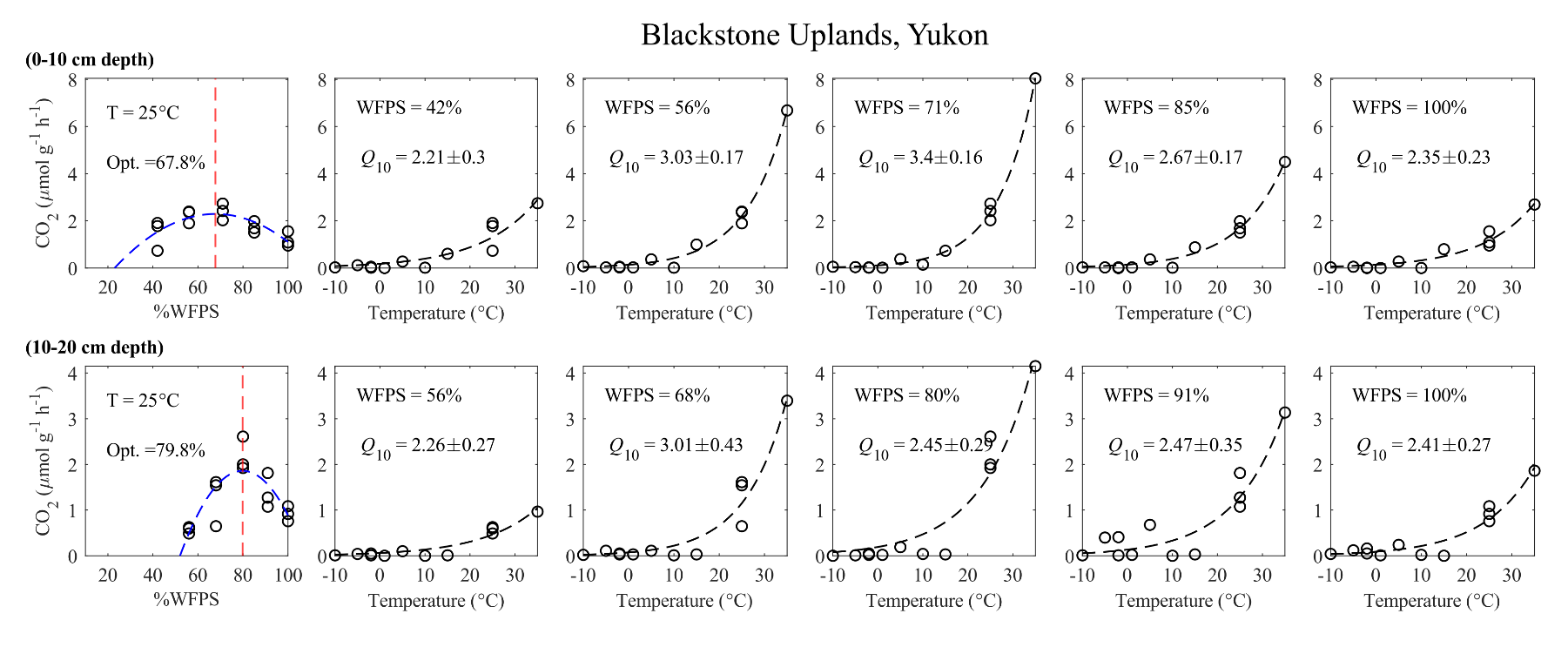


**Figure S3:** CO_2_ production rates measured in peat samples from Blackstone Uplands (see Table 1 for the site information) at different moisture contents (%WFPS) and fitted with Equation (1) and Equation (5) in the main text. The effect of moisture variation at a fixed temperature (25°C) incubation is shown in the first leftmost column with the concave down trend and an optimum moisture level for the maximum CO_2_ production rate. The effects of temperature variations on CO_2_ production at varying peat moisture contents are shown in the next five columns (in increasing order of %WFPS) to derive *Q*_10_ values.


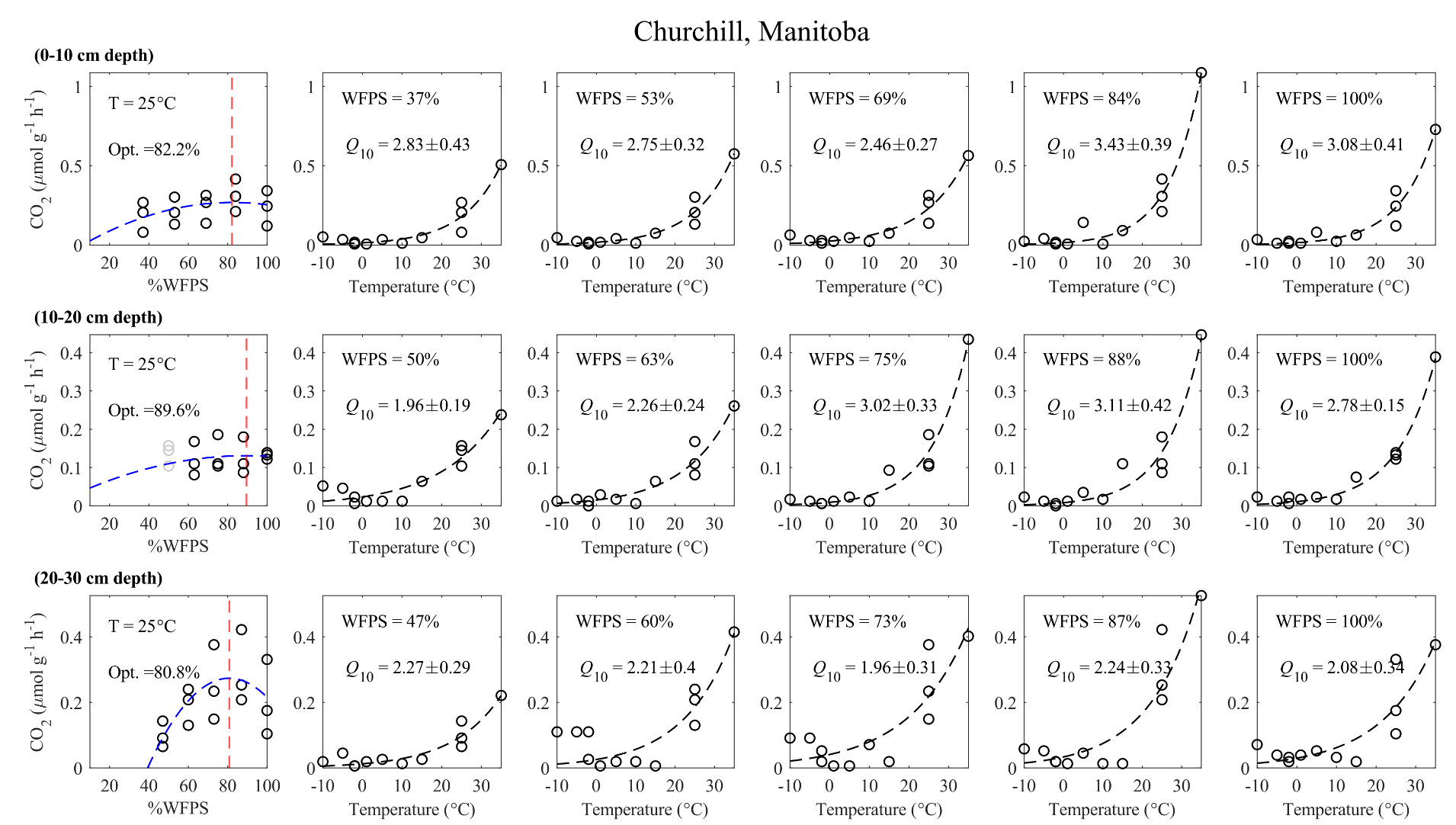


**Figure S4:** CO_2_ production rates measured in peat samples from Churchill site (see Table 1 for the site information) at different moisture contents (%WFPS) and fitted with Equation (1) and Equation (5) in the main text. The effect of moisture variation at a fixed temperature (25°C) incubation is shown in the first leftmost column with the concave down trend and an optimum moisture level for the maximum CO_2_ production rate. The effects of temperature variations on CO_2_ production at varying peat moisture contents are shown in the next five columns (in increasing order of %WFPS) to derive *Q*_10_ values.


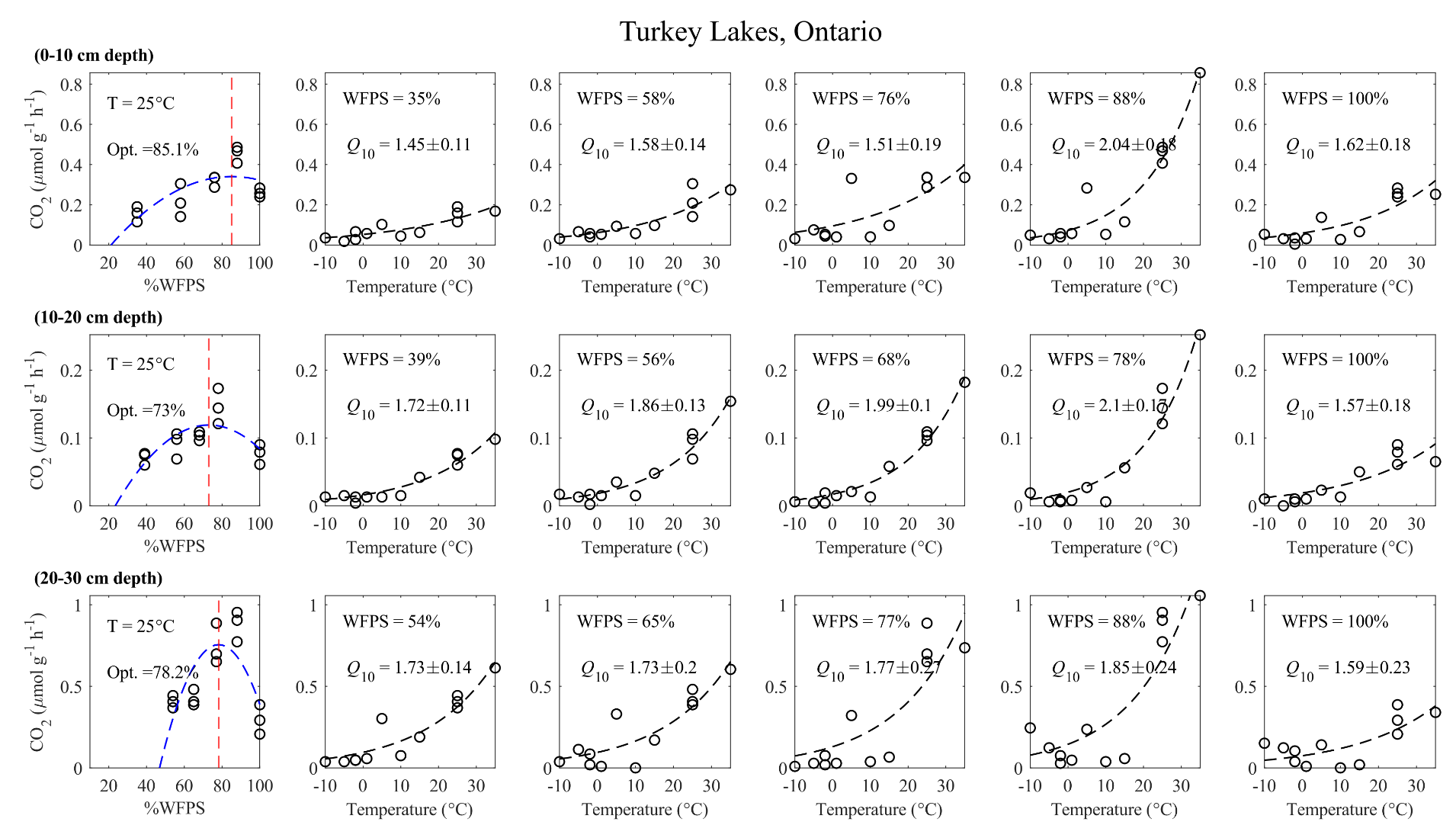


**Figure S5:** CO_2_ production rates measured in peat samples from Turkey Lakes site (see Table 1 for the site information) at different moisture contents (%WFPS) and fitted with the Equation (1) and Equation (5) in the main text. The effect of moisture variation at a fixed temperature (25°C) incubation is shown in the first leftmost column with the concave down trend and an optimum moisture level for the maximum CO_2_ production rate. The effects of temperature variations on CO_2_ production at varying peat moisture contents are shown in the next five columns (in increasing order of %WFPS) to derive *Q*_10_ values.


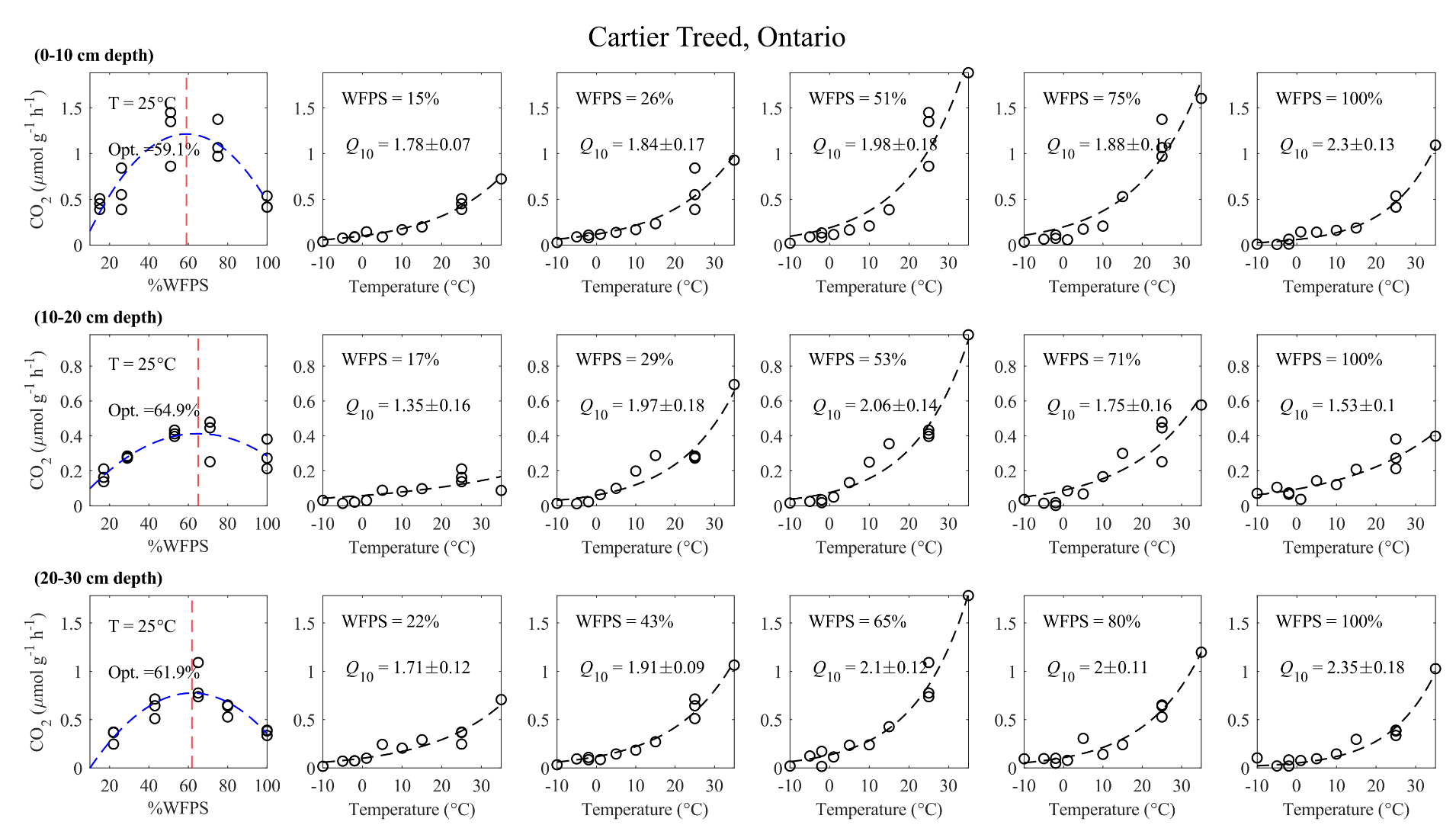


**Figure S6:** CO_2_ production rates measured in peat samples from Cartier Treed peatland site (see Table 1 for the site information) at different moisture contents (%WFPS) and fitted with Equation (1) and Equation (5) in the main text. The effect of moisture variation at a fixed temperature (25°C) incubation is shown in the first leftmost column with the concave down trend and an optimum moisture level for the maximum CO_2_ production rate. The effects of temperature variations on CO_2_ production at varying peat moisture contents are shown in the next five columns (in increasing order of %WFPS) to derive *Q*_10_ values.


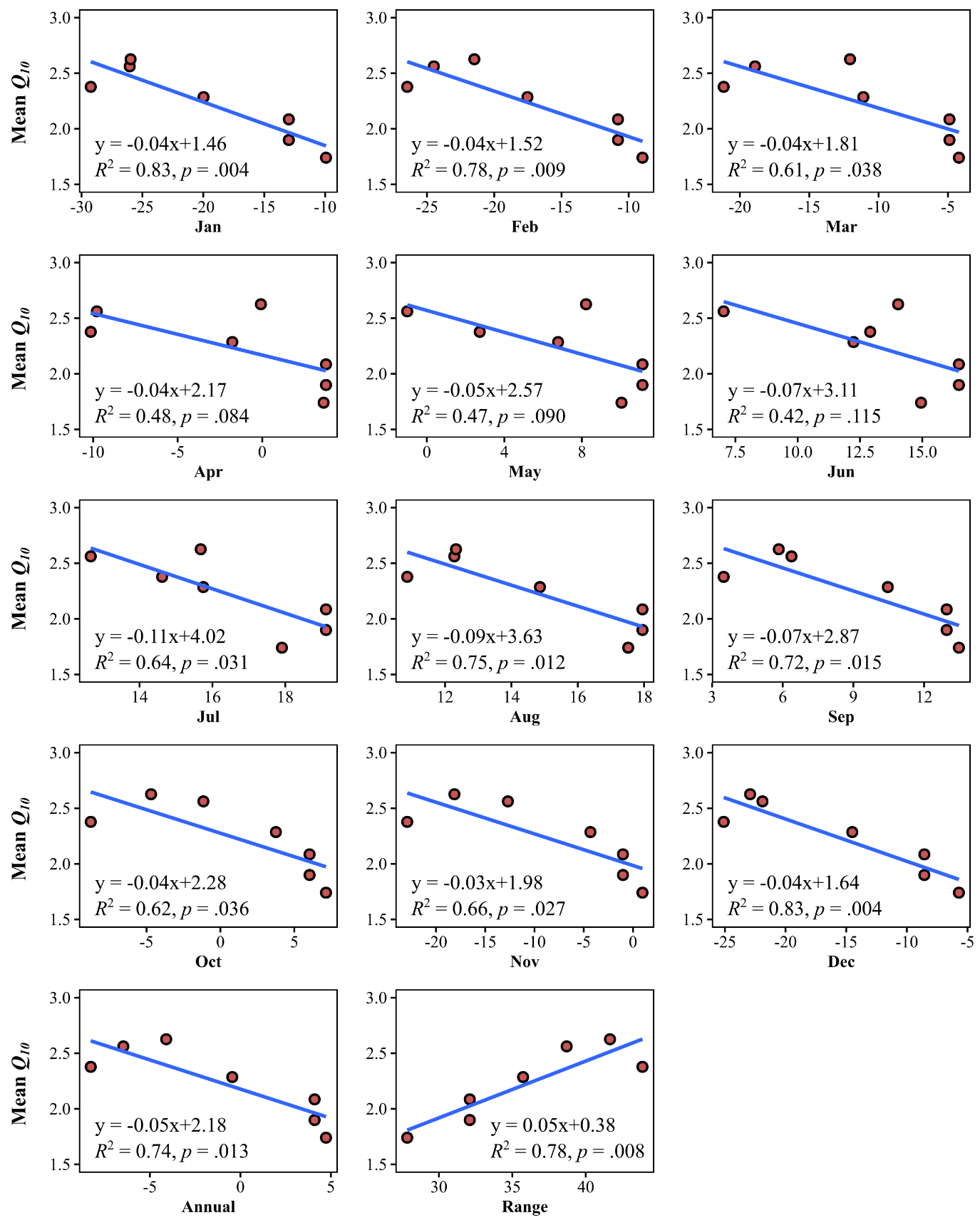


**Figure S7:** Simple linear regression models for the site *Q*_10_ average values by the site mean air temperatures (°C), from every month (Jan, Feb, …, and Dec) to annual mean (Annual). The annul air temperature range (Range) is the difference between July (warmest) and January (coldest) mean temperatures. Performed in R (version 4.0.3).


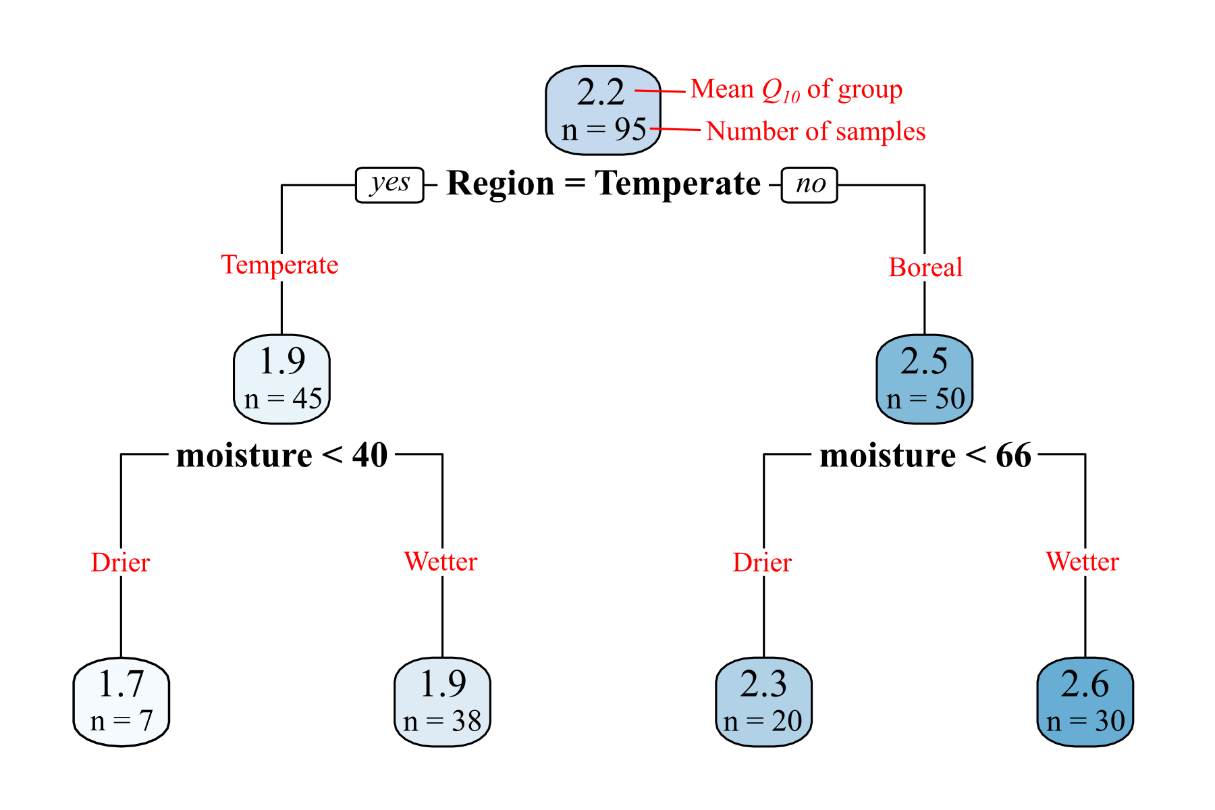


**Figure S8:** A decision tree learning approach to identify the relative importance of site ecoclimate, peat depth, and moisture content (%WFPS) variations in the prediction of the fitted *Q*_10_ value for each peat sample incubation (Figure 2 and Supplementary Figures S2-6). Analysis performed in R 4.0.3 (see the code script and results in the box below).

| > tbl <- read.csv(‘Q10table.csv’) # ‘Q10table.csv’ in supplementary data  > q10tree <- rpart(Q10~Depth+moisture+Region, data = tbl, method = "anova")  > printcp(q10tree)  Regression tree:  rpart(formula = Q10 ~ Depth + moisture + Region, data = tbl,  method = "anova")  Variables actually used in tree construction:  [1] moisture Region  Root node error: 18.152/95 = 0.19107  n= 95  CP nsplit rel error xerror xstd  1 0.389816 0 1.00000 1.02545 0.153190  2 0.076154 1 0.61018 0.63596 0.091686  3 0.022229 2 0.53403 0.57755 0.082786  4 0.010000 3 0.51180 0.60811 0.089036  > q10tree$variable.importance  Region moisture Depth  7.0759413 2.1003349 0.7862157 |
| --- |

**Table S1**: Chemical compositions of the soil water sampled in field and the artificial water solution prepared in laboratory to adjust moisture contents. Ion concentration measured in millimole per litre (mM).

|  | Old Crow Flats | | Blackstone Uplands | | Churchill | | James Bay Bog | | Turkey Lakes | | Cartier Treed | | Cartier Lawn | |
| --- | --- | --- | --- | --- | --- | --- | --- | --- | --- | --- | --- | --- | --- | --- |
|  | Field | Lab | Field | Lab | Field | Lab | Field | Lab | Field | Lab | Field | Lab | Field | Lab |
| Na^+^ | 0.1443 | 0.1443 | 0.1507 | 0.1507 | 0.6151 | 0.3908 | 0.1724 | 0.1150 | 0.1384 | 0.1384 | 0.6371 | 0.6208 | 0.3462 | 0.2810 |
| Ca^2+^ | 0.5207 | 0.0230 | 0.1081 | 0.0700 | 0.0561 | 0.0561 | 0.0263 | 0.0263 | 1.9400 | 1.2173 | 0.0691 | 0.0450 | 0.0267 | 0.0267 |
| Mg^2+^ | 0.4266 | 0.4000 | 0.1376 | 0.0467 | 0.0392 | 0.0395 | 0.0106 | 0.0106 | 0.2717 | 0.2717 | 0.0437 | 0.0437 | 0.0207 | 0.0207 |
| K^+^ | 3.4567 | 3.5950 | 0.7509 | 0.7190 | 0.0768 | 0.0129 | 0.4154 | 0.4174 | 0.1026 | 0.0813 | 4.2802 | 3.7638 | 0.6127 | 0.4887 |
| Cl^-^ | 0.4973 | 3.9543 | 0.4028 | 0.3907 | 0.3343 | 0.3353 | 0.1141 | 0.1476 | 0.0813 | 0.0813 | 2.2157 | 2.2157 | 0.5432 | 0.6034 |
| SO_4_^2-^ | 0.2438 | 0.2430 | 0.0467 | 0.0467 | 0.0565 | 0.1521 | 0.0301 | 0.0306 | 1.5854 | 1.5854 | 0.6208 | 0.7095 | 0.1524 | 0.1517 |
| NO_3_^-^ | 0.0124 | 0 | NA | 0 | 0.0129 | 0.0129 | 0.0194 | 0.0194 | 0.0843 | 0.0840 | 0.0481 | 0.0481 | 0.0887 | 0.0887 |
| PO_4_^-3^ | 0.5917 | 0.5800 | 0.0770 | 0.0760 | NA | 0 | NA | 0 | NA | 0 | 0.3899 | 0.4000 | 0.0132 | 0 |

**Table S2**: Peatland site air temperature data for the simple linear regression analysis for the site mean *Q*_10_ values. Detailed site information in the main text Table 1. Climate stations and data from Canadian Climate Normals (1981-2010) available through Government of Canada open data.

| **Site** | **Climate station^*^** | **Mean *Q*_10_** | **Jan** | **Feb** | **Mar** | **Apr** | **May** | **Jun** | **Jul** | **Aug** | **Sep** | **Oct** | **Nov** | **Dec** | **Annual** | **Range**  **(Jul - Jan)** |
| --- | --- | --- | --- | --- | --- | --- | --- | --- | --- | --- | --- | --- | --- | --- | --- | --- |
| Old Crow Flats | OLD CROW A | 2.38 | -29.24 | -26.47 | -21.17 | -10.14 | 2.73 | 12.91 | 14.62 | 10.86 | 3.49 | -8.77 | -22.92 | -25.1 | -8.27 | 43.86 |
| Blackstone Uplands | DAWSON A | 2.63 | -25.97 | -21.48 | -12.06 | -0.08 | 8.21 | 14.03 | 15.68 | 12.33 | 5.83 | -4.69 | -18.13 | -22.92 | -4.1 | 41.65 |
| Churchill | CHURCHILL A | 2.56 | -26.04 | -24.49 | -18.91 | -9.78 | -1.01 | 7.03 | 12.66 | 12.28 | 6.37 | -1.15 | -12.7 | -21.9 | -6.47 | 38.7 |
| James Bay Bog | MOOSONEE UA | 2.29 | -19.98 | -17.53 | -11.1 | -1.77 | 6.77 | 12.24 | 15.75 | 14.86 | 10.46 | 3.75 | -4.32 | -14.5 | -0.45 | 35.73 |
| Turkey Lakes | SAULT STE MARIE A | 1.74 | -9.93 | -8.98 | -4.21 | 3.63 | 10.04 | 14.95 | 17.91 | 17.52 | 13.48 | 7.14 | 0.96 | -5.71 | 4.73 | 27.84 |
| Cartier Treed | SUDBURY A | 1.90 | -12.98 | -10.8 | -4.89 | 3.77 | 11.12 | 16.47 | 19.12 | 17.95 | 12.96 | 6.03 | -1.02 | -8.56 | 4.1 | 32.1 |
| Cartier Lawn | SUDBURY A | 2.09 | -12.98 | -10.8 | -4.89 | 3.77 | 11.12 | 16.47 | 19.12 | 17.95 | 12.96 | 6.03 | -1.02 | -8.56 | 4.1 | 32.1 |

*Find details online (<https://climate.weather.gc.ca/climate_normals/index_e.html>) by searching the name of climate station as in this column and check matching climate dataset with climate identifier (ID) as follows: OLD CROW A (ID 2100800); DAWSON A (ID 2100402); CHURCHILL A (ID 5060600); MOOSONEE UA (ID 6075425); SAULT STE MARIE A (ID 6057592); SUDBURY A (ID 6068150).
